# Directing the Linkage of Small Polyoxometalate Building Blocks Using (Benzene)ruthenium Cations

**DOI:** 10.1021/acs.inorgchem.4c04150

**Published:** 2024-12-10

**Authors:** Sugiarto, Yuudai Iwai, Ryo Ohtani, Masahiro Sadakane

**Affiliations:** † Department of Applied Chemistry, Graduate School of Advanced Science and Engineering, Hiroshima University, 1-4-1 Kagamiyama, Higashi-Hiroshima 739-8527, Japan; ‡ Department of Chemistry, Faculty of Science, Kyushu University, 744 Motooka, Nishi-ku, Fukuoka 819-0395, Japan

## Abstract

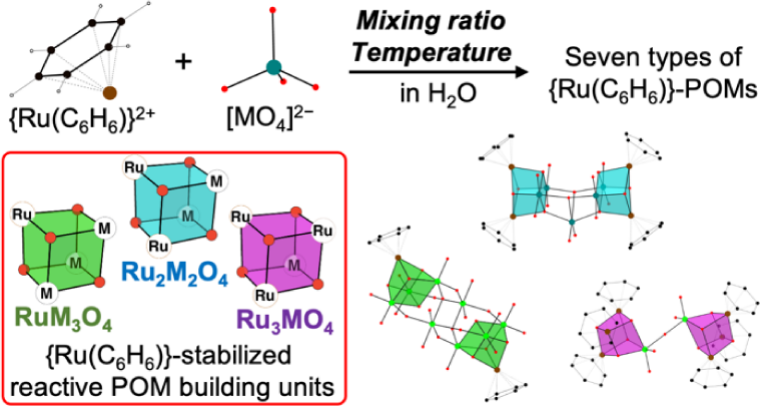

Oligomerization of monomeric molybdate and tungstate oxyanions in the presence of organometallic cations produces a group of organometallic-polyoxometalate clusters, which have diverse structures based on cubane-like {M_4_O_4_} units resembling the structure of oxide surfaces. This work investigated the oligomerization of [MoO_4_]^2–^ and [WO_4_]^2–^ oxyanions in aqueous solutions in the presence of {Ru(C_6_H_6_)}^2+^ as the organometallic structure-directing agents. The reactions produced a mixture of several species, and fractional crystallization by adjusting crystallization temperature and molar ratios of Ru:Mo or Ru:W allowed the isolation of seven types of (benzene)ruthenium–polyoxometalate complexes: [{Ru(C_6_H_6_)}_4_Mo_4_O_16_] (**1**), [{Ru(C_6_H_6_)(OH)}_3_HMoO_4_][MoO_4_] (**2**), [{Ru(C_6_H_6_)}_4_H_2_Mo_5_O_20_] (**3**), [{Ru(C_6_H_6_)}_4_W_2_O_10_] (**4**), Na_6_[{Ru(C_6_H_6_)}_2_H_2_W_8_O_30_] (**5**), [{Ru(C_6_H_6_)}_5_H_2_W_6_O_24_] (**6**), and [{Ru(C_6_H_6_)(OH)}_6_W_2_O_7_][{Ru(C_6_H_6_)}_2_{Ru(C_6_H_6_)(OH_2_)}_2_H_2_W_8_O_30_]_2_ (**7**). They comprise small, reactive *iso*-polyoxometalate building blocks that are capped by {Ru(C_6_H_6_)}^2+^ cations, resulting in cubane-like {Ru_3_MO_4_}, {Ru_2_M_2_O_4_}, or {RuM_3_O_4_} (M = Mo or W) structural motifs. Moreover, the molecular crystal of **1** contains extensive C–H···O hydrogen bonds, and it undergoes a reversible crystal-to-amorphous-to-crystal transition upon dehydration–hydration cycles.

## Abstract 2

We report the synthesis of (benzene)ruthenium−polyoxometalate complexes formed in the reaction mixture of [{Ru(C_6_H_6_)Cl}_2_(μ-Cl)_2_] and Na_2_[MoO_4_] or Na_2_[WO_4_] in water. This work demonstrates the ability of organoruthenium complexes to stabilize reactive polyoxometalate anions and guide the linkage of metal−oxygen clusters into new structures and functions.

## Introduction

**Scheme 1:**
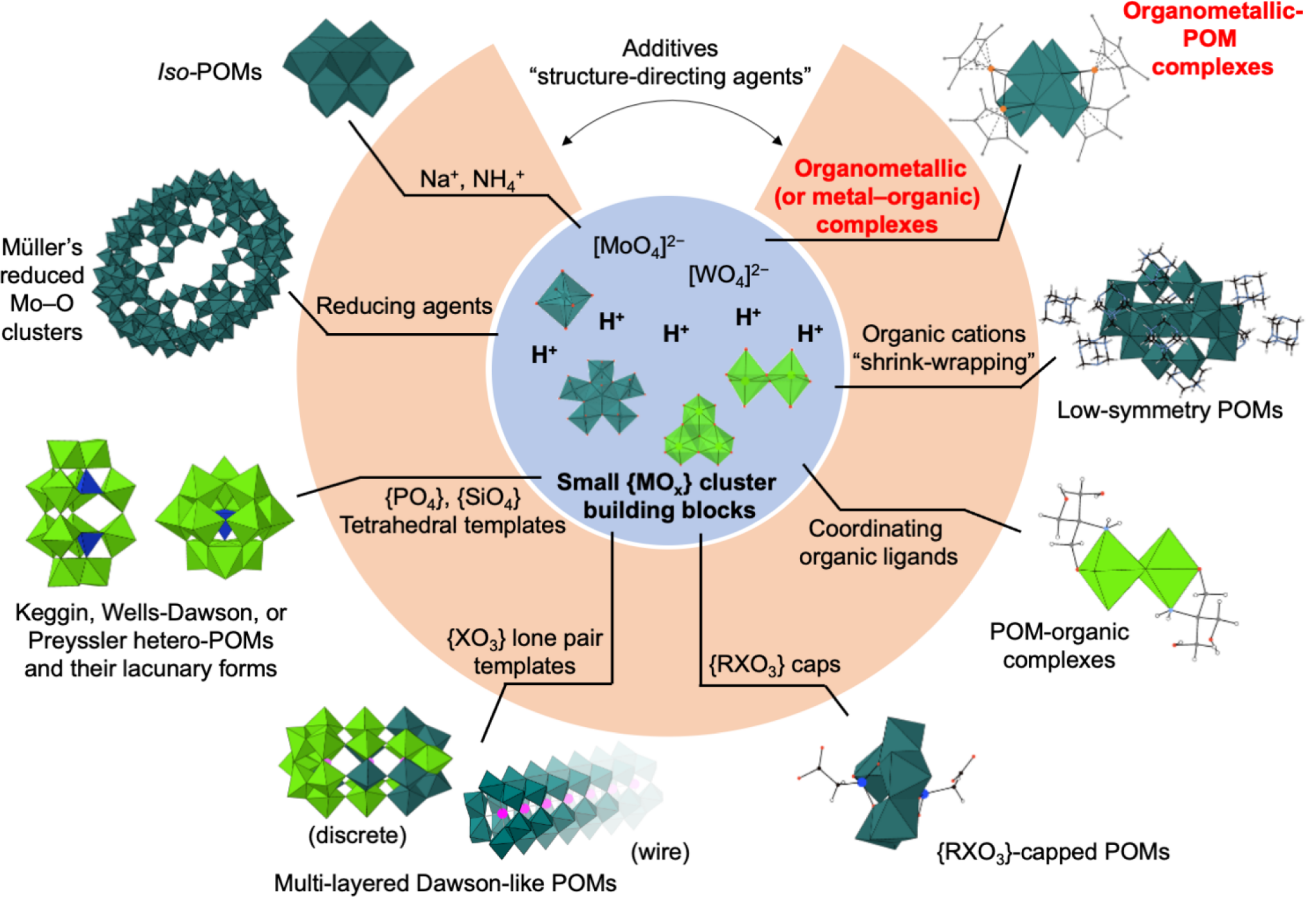
Common Additives for POM Syntheses in Aqueous Media Selected examples (clockwise from top right): organoiridium-POM [{IrC_5_Me_5_}_4_Mo_4_O_16_],^7^ HMTAH-wrapped [H_2_Mo^VI^_12_Mo^V^_4_O_52_]^10–^ (HMTAH = protonated hexamethylenetetramine),^8^ tris-stabilized {W_2_O_6_} dimer,^9^ phosphonocarboxylate-capped {Mo_5_O_15_},^10^ discrete^11^ and wire^12^ multi-layered Dawson-like POM containing {XO_3_} groups, Keggin and Wells–Dawson POMs and their lacunary clusters, Müller’s {Mo_154_} big wheel,^13^ and [Mo_7_O_24_]^6–^.

Molecular metal oxide anions of V, Nb, Ta, Mo, and W (widely known as polyoxometalates, POMs) are products of controlled hydrolysis–condensation reactions of the mononuclear oxyanions (VO_4_^3–^, MoO_4_^2–^, and WO_4_^2–^) in acidic conditions^1^ and have diverse structures ranging from dinuclear clusters such as [Mo_2_O_7_]^2–^ to Müller’s massive molybdenum oxide clusters such as [H_16_Mo_368_O_1032_(H_2_O)_240_(SO_4_)_48_]^48–^.^2−4^ The structure of POMs is constructed by the linkage of small {M*_x_*O*_y_*} metal–oxo building blocks,^5^ and exploration of new POMs requires additional ingredients (in addition to the oxyanion precursors and acids) as structure-directing agents to generate unique building units and control their linkage into metal–oxygen backbones. For example, Müller’s [H_16_Mo_368_O_1032_(H_2_O)_240_(SO_4_)_48_]^48–^ comprises {MoO}, {Mo_2_O_5_}, and pentagonal {Mo_6_O_21_} units, and their synthesis requires Na_2_S_2_O_4_ and SO_4_^2–^ additives as the reducing agent and coordinating anion, respectively.^6^Scheme 1 summarizes selected examples of additives for POM syntheses in aqueous media.

As shown in Scheme 1, organometallic cations such as {RhC_5_Me_5_}^2+^ and {Ru(arene)}^2+^ function as structure-directing agents that drive the oligomerization of VO_4_^3–^, MoO_4_^2–^, or WO_4_^2–^ anions into organometallic-POM complexes having cubane-like {M_4_O_4_} building blocks.^7,14,15^ Organometallic POMs have attracted attention because of their structural relevance to the surfaces of heterogeneous catalysts,^16^ their dynamic metal–oxygen backbone due to the flexible coordination bonds between the organometallic cations and the POM surfaces,^17−21^ and their application as molecular precursors for solid catalysts with high metal/oxide interfaces.^22,23^ More importantly, this synthetic method allows stabilization of small and reactive POMs such as [Mo_4_O_16_]^8–^ and easy tuning of the metal–oxygen structure by adjusting the charge of organometallic cations, the size of organic ligands, and the number of metal’s coordination sites.^24^ For example, the reaction of [{Ru(arene)Cl}_2_(μ-Cl)_2_] and Na_2_[MoO_4_] in a 1:5 Ru:Mo mixing ratio produces, depending on the type of the arene ligand, a windmill-shaped [{Ru(*p*-cymene)_4_}Mo_4_O_16_]^25^ or a monolacunary Lindqvist [{Ru(C_6_Me_6_)}_2_{Ru(C_6_Me_6_)(OH_2_)}Mo_5_O_18_] cluster.^26^

While many work focuses on the reactivity of bulkier organoruthenium cations such as {Ru(*p*-cymene)}^2+^ and {Ru(hexamethylbenzene)}^2+^ with mononuclear VO_4_^3–^, MoO_4_^2–^, and WO_4_^2–^ anions,^25−28^ there is a need to clarify the reactivity of the least bulky {Ru(benzene)}^2+^ with the monomeric oxometalate anions because previous studies show that the reaction is tricky. Bi et al. reported that KNa[{Ru(C_6_H_6_)}_2_(CH_3_COO)_6_] reacts with Na_2_[WO_4_] in a 1:1.875 Ru:W mixing ratio, producing [{Ru(C_6_H_6_)}_2_H_2_W_8_O_30_]^6–^ after one-month crystallization.^29^ They found KNa[{Ru(C_6_H_6_)}_2_(CH_3_COO)_6_] is crucial to obtain [{Ru(C_6_H_6_)}_2_H_2_W_8_O_30_]^6–^ as the reaction of [{Ru(C_6_H_6_)Cl}_2_(μ-Cl)_2_] and Na_2_[WO_4_] gives [{Ru(C_6_H_6_)}_2_{Ru(C_6_H_6_)(OH_2_)}_2_H_2_W_8_O_30_]^2–^, which has a similar structure to the *p*-cymene analogue reported by Proust’s group.^26^ Abramov et al. reported a faster synthetic method for [{Ru(C_6_H_6_)}_2_H_2_W_8_O_30_]^6–^ by the reaction of [Ru(C_6_H_6_)(OH_2_)_3_]^2+^ and Na_2_[WO_4_] in a 1:3 Ru:W mixing ratio in the presence of SeO_2_ to improve the reaction yield, although selenium is not present in the target compound.^30^ To the best of our knowledge, [{Ru(C_6_H_6_)}_2_H_2_W_8_O_30_]^6–^ is the only well-characterized complex of {Ru(C_6_H_6_)}^2+^ with *iso*-POMs. Note that there are several complexes of {Ru(C_6_H_6_)}^2+^ with lacunary *hetero*-POMs such as [{Ru(C_6_H_6_)}(γ-SiW_10_O_36_)];^31−34^ this type of cluster is not the focus of the current paper.

**Scheme 2:**
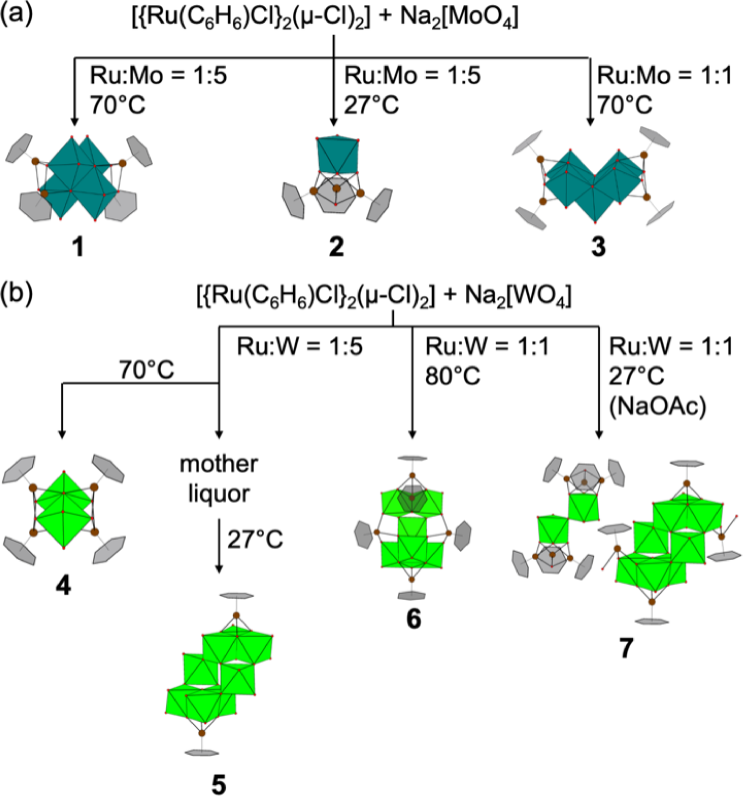
Reaction Conditions for the Isolation of (a) (C_6_H_6_)Ru–Polyoxomolybdate and (b) (C_6_H_6_)Ru–Polyoxotungstate Complexes NaOAc is sodium acetate. Ru:Mo or Ru:W indicates the molar ratio between the *total* amount of Ru and Mo (or W).

Recently, we found that the mixing ratio of the organometallic and oxometalate precursors controls the speciation of {IrC_5_Me_5_}–polyoxotungstate clusters.^35^ As such, we were interested in whether our synthetic concept is applicable to scrutinize the speciation of {Ru(C_6_H_6_)}^2+^-coordinated *iso*-POMs. Herein, we show that the reaction of [{Ru(C_6_H_6_)Cl}_2_(μ-Cl)_2_] and Na_2_[MoO_4_] or Na_2_[WO_4_] in aqueous solution produced seven types of (C_6_H_6_)Ru-POM complexes: [{Ru(C_6_H_6_)}_4_Mo_4_O_16_] (**1**), [{Ru(C_6_H_6_)(OH)}_3_HMoO_4_][MoO_4_] (**2**), [{Ru(C_6_H_6_)}_4_H_2_Mo_5_O_20_] (**3**), [{Ru(C_6_H_6_)}_4_W_2_O_10_] (**4**), Na_6_[{Ru(C_6_H_6_)}_2_H_2_W_8_O_30_] (**5**), [{Ru(C_6_H_6_)}_5_H_2_W_6_O_24_] (**6**), and a cation–anion pair [{Ru(C_6_H_6_)(OH)}_6_W_2_O_7_][{Ru(C_6_H_6_)}_2_{Ru(C_6_H_6_)(OH_2_)}_2_H_2_W_8_O_30_]_2_ (**7**). These compounds were isolable as crystalline solids by adjustment of the mixing proportion of the reactants and the crystallization temperature, as shown in Scheme 2. Their structure was characterized by single-crystal X-ray diffraction (SCXRD), powder X-ray diffraction (PXRD), Fourier transform infrared (FTIR) spectroscopy, and ^1^H NMR spectroscopy. Their chemical formula was confirmed by elemental and thermogravimetric analysis (TGA).

## Experimental Section

### Materials and Analytical Methods

[{Ru(C_6_H_6_)Cl}_2_(μ-Cl)_2_] (Sigma-Aldrich), Na_2_[MoO_4_]·2H_2_O (FUJIFILM Wako Pure Chemical Industries), Na_2_[WO_4_]·2H_2_O (Nippon Inorganic Color and Chemical), and Na(CH_3_COO) (FUJIFILM Wako Pure Chemical Industries) were used without further purification. Distilled water for synthesis was produced using an Elix Essential UV 3 water purifying system. The pH of the solution was measured was using a Horiba D-54 pH meter equipped with a LAQUA 9618S-10D Micro ToupH electrode; all solutions were cooled down to room temperature (20–22 °C) before pH measurement. FTIR spectra were collected using a JASCO 4X spectrometer. PXRD patterns at ambient conditions were collected using a Bruker D2 Phaser second Generation equipped with a Cu *K*α (λ = 1.54184 Å) radiation source and a 1D LYNXEYE detector, whereas variable temperature PXRD patterns of **1** were recorded under a nitrogen flow on a Rigaku MiniFlex600 equipped with an Anton Paar heating device (BTS 500), and samples after dehydration at 100 °C using a vacuum pump (max. vacuum: approximately 0.067 Pa) were packed with an airtight sample holder in a dry glovebox. ^1^H NMR spectra were recorded on a Variant System 500 (500 MHz; H resonance frequency = 499.827 MHz) spectrometer at ambient temperature, and the chemical shifts were referred to the residual signal of the solvent. Thermogravimetric (TG) analysis was carried out using a Hitachi SII TG/DTA7300 analyzer with a heating rate of 10 °C/min under a constant air flow (200 mL/min). Water vapor, nitrogen, and carbon dioxide sorption measurements were performed at 25, −196, and 25 °C, respectively, on a Belsorb MAX (Bel, Japan) apparatus; the sample was evacuated at 100 °C for 2 h under a dynamic vacuum before adsorption measurement. Total elemental (except for oxygen) analyses of all compounds were carried out by Mikroanalytisches Labor Pascher (Remagen, Germany).

### Synthesis of [{Ru(C_6_H_6_)}_4_Mo_4_O_16_]·*n*H_2_O (**1**)

A mixture of [{Ru(C_6_H_6_)Cl}_2_(μ-Cl)_2_] (25.0 mg; 0.05 mmol) and Na_2_[MoO_4_]·2H_2_O (120.9 mg; 0.5 mmol) in water (1.0 mL) was dissolved at 80 °C under stirring for 2 min. The resulting clear brown solution (pH = 6.7) was filtered while hot through cotton wool and set up for crystallization in a sealed vial in a drying oven (70 °C) overnight. The well-formed reddish-brown blocks were collected by suction filtration, rinsed with water under suction (the crystals were insoluble in water) until the effluent became colorless, and dried in the air. The pH of the mother liquor was 6.8. Yield = 13.1 mg (35% based on Ru). FTIR (KBr, 950–450 cm^–1^ region): 930, 895, 855, 701, 652, 611, 591, 497, 472. Elem. anal. found: C, 19.06; H, 2.6; Cl, <0.1; Mo, 25.8; Na, 0.07; Ru, 27.6. Calcd for *n* = 7: C, 19.44; H, 2.59; Cl, 0; Mo, 25.89; Na, 0; Ru, 27.27. TG analysis (see the thermogram in Figure S1) until 150 °C found a 7.93% weight decrease corresponding to the loss of the water solvates (calcd 7.95% for *n* = 6.5).

### Synthesis of [{Ru(C_6_H_6_)(OH)}_3_HMoO_4_][MoO_4_]·*n*H_2_O (**2**)

A mixture of [{Ru(C_6_H_6_)Cl}_2_(μ-Cl)_2_] (25 mg; 0.05 mmol) and Na_2_[MoO_4_]·2H_2_O (121.1 mg; 0.5 mmol) in water (1.0 mL) was dissolved with stirring at room temperature (20–22 °C) for 30 min. The resulting brown solution was evaporated to dryness at 27 °C in an incubator; this evaporation process took approximately 1 week. The remaining black residues were redissolved in water (0.3 mL), and the viscous solution was passed through cotton wool to remove brown crystals of **1**. The filtrate was left at 20–22 °C in an open vial to deposit (within hours) yellow crystals of **2**. After overnight crystallization, the crystals were collected by suction filtration, rinsed with several drops of ice-cooled water, and dried in the air. Yield = 9.6 mg (28% based on Ru). FTIR (ν in the 950–450 cm^–1^ region): 924, 897, 861, 818, 785, 622, 608, 517, 474. Elem. anal. found: C, 21.05; H, 3.18; Cl, 0.26; Mo, 19; Na, 0.25; Ru, 29.6. Calcd for *n* = 6 and 0.1 mol of NaCl contaminant: C, 21.12; H, 3.35; Cl, 0.35; Mo, 18.75; Na, 0.22; Ru, 29.63. TG analysis (Figure S1) until 430 °C found a 34.05% decrease in weight corresponding to the decomposition of **2** to 3RuO_2_·2MoO_3_ (calcd 34.02% for *n* = 7).

### Synthesis of [{Ru(C_6_H_6_)}_4_H_2_Mo_5_O_20_]·*n*H_2_O (**3**)

[{Ru(C_6_H_6_)Cl}_2_(μ-Cl)_2_] (25.0 mg; 0.05 mmol) was dissolved in water (4.0 mL) at 80 °C under stirring for approximately 6 min, and Na_2_[MoO_4_]·2H_2_O (24.2 mg; 0.1 mmol) was added in one portion to the hot solution. Precipitation occurred immediately, and the mixture was stirred for another 1 min and filtered while hot through a disposable membrane filter (Advantec, DISMIC = 0.2 μm). The resulting clear brown solution was sealed in a glass vial and set in a drying oven at 70 °C for crystallization. Many microcrystals formed within minutes, and crystallization was continued overnight. The crystals were harvested using suction filtration, rinsed with water (the crystals were insoluble in water) under suction until the effluent became colorless, and dried in air. Yield = 13.5 mg (33% based on Ru). FTIR (ν in the 950–450 cm^–1^ region): 930, 896, 849, 744, 658, 609, 547, 535, 505, 479. Elem. anal. found: C, 16.83; H, 2.27; Cl, <0.03; Mo, 28.6; Na, 0.04; Ru, 25.2. Calcd for *n* = 7: C, 17.52; H, 2.46; Cl, 0; Mo, 29.17; Na, 0; Ru, 24.59. Note that the amount of Ru found was higher than that calculated based on the chemical formula of **3**; however, the results of the elemental analyses fit well if 0.15 mol RuO_2_ (with *n* = 7.5) was included as a contaminant: C, 17.22; H, 2.47; Cl, 0; Mo, 28.67; Na, 0; Ru, 25.07. TG analysis (Figure S1) until 200 °C found a 7.99% weight decrease corresponding to the loss of the water solvates (calcd 8.07% for *n* = 7.5).

### Synthesis of [{Ru(C_6_H_6_)}_4_W_2_O_10_]·*n*H_2_O (**4**)

A mixture of [{Ru(C_6_H_6_)Cl}_2_(μ-Cl)_2_] (50.0 mg; 0.1 mmol) and Na_2_[WO_4_]·2H_2_O (329.9 mg; 1.0 mmol) in water (2.0 mL) was stirred on a hot plate at 80 °C for 5 min. The clear dark brown solution (pH = 7.63, measured at room temperature) was filtered while hot to remove a few undissolved solid. The clear filtrate was transferred into a glass vial, sealed, and set in a drying oven (70 °C) for crystallization. Well-developed red-brown block-shaped crystals of **4** were deposited within hours, and crystallization was continued overnight (approximately 19 h). The hot solution was cooled to room temperature, and reddish-brown crystals of **4** were harvested by filtration and washed with water (the crystal was insoluble in water) until the effluent became colorless. Yield = 18.9 mg (27% based on Ru). FTIR (KBr, 950–450 cm^–1^ region): 919, 899, 878, 842, 647, 612, 590, 510, 475. Elem. anal. found: C, 19.96; H, 3.12; Cl, <0.1; Na, 0.03; Ru, 28.8; W, 25.4. Calcd for *n* = 10: C, 20.23; H, 3.12; Cl, 0; Na, 0; Ru, 28.39; W, 25.8. TG analysis (Figure S2) until 150 °C found a 12.9% weight decrease corresponding to the loss of the water solvates (calcd 12.65% for *n* = 10).

### Synthesis of Na_6_[{Ru(C_6_H_6_)}_2_H_2_W_8_O_30_]·*n*H_2_O (**5**)

The remaining mother liquor after harvesting the crystals of **4** (pH = 7.74) was allowed to evaporate in an open vial at room temperature to deposit yellow rods of **5**. After 4–5 days, crystals of **5** were harvested by filtration and washed with few drops of ice-cold water under suction to remove excess mother liquor. Yield = 42.5 mg (16% based on Ru). FTIR (KBr, 950–450 cm^–1^ region): 929, 882, 811, 755, 669, 587, 565, 525, 458. Elem. anal. found: C, 5.27; H, 1.96; Cl, <0.1; Na, 4.68; Ru, 7.78; W, 51. Calcd for *n* = 22 with 0.08 mol of [{Ru(C_6_H_6_)}_2_(μ–OH)_3_]Cl contaminant: C, 5.40; H, 2.08; Cl, 0.1; Na, 4.79; Ru, 7.58; W, 51.04. TG analysis (Figure S2) until 300 °C found a 12.16% weight decrease corresponding to the loss of the water solvates (calcd 12.11% for *n* = 19).

### Synthesis of [{Ru(C_6_H_6_)}_5_H_2_W_6_O_24_]·*n*H_2_O (**6**)

A mixture of [{Ru(C_6_H_6_)Cl}_2_(μ-Cl)_2_] (25 mg; 0.05 mmol) and Na_2_[WO_4_]·2H_2_O (33 mg; 0.1 mmol) in water (2.0 mL) was dissolved at 80 °C under stirring. The resulting cloudy solution was filtered while hot through a disposable membrane filter (Advantec, DISMIC 0.2 μm), and the filtrate was crystallized on a hot plate (80 °C) in a sealed vial. Well-developed brown crystals were harvested after 2–3 h, washed with 3 drops of cold water, and air-dried. Yield = 10.3 mg (19% based on Ru). FTIR (KBr, 950–450 cm^–1^ region): 932, 881, 841, 729, 669, 615, 583, 540, 469. Elem. anal. found: C, 13.03; H, 2.59; Cl, <0.1; Na, 0.11; Ru, 19.3; W, 40.5 Calcd for *n* = 17 with 0.08 mol [{Ru(C_6_H_6_)}_2_(μ-OH)_3_]Cl and 0.05 mol Na_2_[WO_4_] contaminant: C, 13.56; H, 2.48; Cl, 0.1; Na, 0.08; Ru, 19.03; W, 40.56. TG analysis (Figure S2) until 200 °C found an 11.28% weight decrease corresponding to the loss of the water solvates (calcd 11.17% for *n* = 17).

### Synthesis of [{Ru(C_6_H_6_)(OH)}_6_W_2_O_7_][{Ru(C_6_H_6_)}_2_{Ru(C_6_H_6_)(OH_2_)}_2_H_2_W_8_O_30_]_2_·*n*H_2_O (**7**)

[{Ru(C_6_H_6_)Cl}_2_(μ-Cl)_2_] (25 mg; 0.05 mmol) and Na_2_[WO_4_]·2H_2_O (33 mg; 0.1 mmol) in 0.1 M Na(CH_3_COO) (2.0 mL) were heated at 80 °C to dissolve. The solution was filtered while hot and set up for crystallization at 27 °C in an open vial. After overnight, a mixture of crystals (brown blocks of **6** and yellow needlelike crystals) was deposited. Crystallization was continued for 1 week, during which more yellow needles were formed. Both types of crystals were harvested by suction filtration through a membrane (filter paper should not be used because the crystals of **7** would stick to the paper), washed with a few drops of water, and air-dried. Yield = 21.5 mg (39% based on Ru). To improve the purity of **7**, crystals of **7** were harvested using a pipet, and some brown crystals of **6** were manually separated under a microscope. FTIR (KBr, 950–450 cm^–1^ region): 925, 875, 817, 748, 591, 526, 472. Elem. anal. found: C, 12.61; H, 2.81; Cl, <0.1; Na, 0.1; Ru, 18.2; W, 39.5. Calcd for *n* = 36 with 0.6 mol of [{Ru(C_6_H_6_)}_2_(μ–OH)_3_]_2_[{Ru(C_6_H_6_)}_2_{Ru(C_6_H_6_)(OH_2_)}_2_H_2_W_8_O_30_]_2_·42H_2_O contaminant: C, 12.8; H, 2.80; Cl, 0; Na, 0; Ru, 17.95; W, 39.58. Note that [{Ru(C_6_H_6_)}_2_{Ru(C_6_H_6_)(OH_2_)}_2_H_2_W_8_O_30_]^2–^ has been reported by Bi et al.,^36^ and a *p*-cymene analogue [{Ru(*p*-cymene)}_2_(μ-OH)_3_]_2_[{Ru(*p*-cymene)}_2_{Ru(*p*-cymene)(OH_2_)}_2_H_2_W_8_O_30_] has been reported by Proust et al.^26^ TG analysis (Figure S2) until 530 °C found a 21.97% weight decrease corresponding to the decomposition of **7** to 14RuO_2_·18WO_3_ (calcd 21.78% for *n* = 36).

### X-ray Crystallography

A single crystal suitable for X-ray diffraction was suspended in mineral oil and mounted on a goniometer head under a stream of cold nitrogen. Intensity data of **5** and **6** were collected at −150 °C on a Bruker SMART APEXII diffractometer equipped with a CCD detector and Mo Kα radiation (λ = 0.71073 Å) source monochromated by layered confocal mirrors. Data reduction, integration, and scaling were performed on a Bruker APEX3 suite.^37^ The intensities were corrected against absorption using SADABS.^37^ Intensity data of **1**–**4** and **7** were collected at −150 °C on a Rigaku XtaLAB Synergy R, DW system equipped with a HyPix Hybrid Pixel Array Detector and Mo Kα radiation (λ = 0.71073 Å) source monochromated by layered confocal mirrors. Data reduction, integration, and scaling were performed on CrysAlisPro. The intensities were corrected against absorption using SCALE3 ABSPACK. The initial structure was determined using the SHELXT^38^ program and subsequently refined using SHELXL^39^ running on a ShelXle^40^ user interface. Some disordered crystallization water was removed using the PLATON SQUEEZE tool.^41^ All of the non-hydrogen atoms were refined anisotropically. The hydrogen atoms of the benzene ligands were generated at the calculated positions using a riding model. The detailed crystallographic parameters are summarized in Table S1.

Crystal structure of **1** was solvable in four different settings: triclinic *P* with *Z*′ = 1, triclinic *P* with *Z*′ = 2, monoclinic *C*2/*c*, and orthorhombic *Fddd*. We collected several sets of diffraction data using different crystals, and the reciprocal lattice clearly showed two domains (major, strong reflections and minor, weak reflections) that could not be fit with any twin laws. We first solved the structure in the triclinic *P* with *Z*′ = 2, and PLATON ADDSYM found possible translational noncrystallographic symmetry. Furthermore, the PLATON CheckCIF report suggested an orthorhombic *Fddd* setting with a 91% fit. Following these suggestions, we solved the structure in orthorhombic *Fddd* in which the asymmetric unit comprised one-fourth of the molecule. Attempts to refine atoms anisotropically gave oxygen atoms of the crystallization water with severely elongated ellipsoids along approximately the [100] direction. Inspection of the crystal packing revealed channels along the [100] direction in which the water solvates were severely disordered. We decided to remove these disordered water molecules using PLATON SQUEEZE.

### Bond Valence Sum (BVS) Calculation

The BVS of Mo, W, and O atoms was calculated using the expression (eq 1) for the variation of length *R*_*ij*_ of a bond between two atoms, *i* and *j*, in an observed crystal with valence *V*_*i*_:^42^
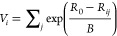
1where *B* is a constant equal to 0.37 Å, and *R*_0_ is the bond valence parameter for a given atom pair; we used *R*_0_ = 1.917 for W^6+^–O^2–^ and 1.907 Å for Mo^6+^–O^2–^ pairs to calculate the BVS. Because *R*_0_ of the Ru^2+^–O^2–^ pair is unknown, the BVS of O atoms binding *simultaneously* to Mo (or W) and Ru was calculated by assuming that the bond valence of a Ru^2+^–O^2–^ bond is 0.4. We chose the value 0.4 by fixing the BVS of μ_3_-OH (O7) in **2** (in which the position of the hydrogen atom was located in the crystal structure) to 1.2. This assumption is reasonable because (i) the BVS of the OH groups binding *solely* to Mo or W atoms in **5** and **7** is approximately 1.20 and (ii) the bond valence of a Ru^2+^–O^2–^ bond (the average of the shortest and longest Ru–O bond lengths in **1**–**7** is 2.07 and 2.13 Å, respectively) calculated using the *R*_0_ for Ru^3+^–O^2–^ (1.77 Å) was in the range of 0.38–0.44. The BVS of the atoms is tabulated in Table S2.

## Results and Discussion

The reaction of [{Ru(C_6_H_6_)Cl}_2_{μ-Cl}_2_] and Na_2_[MoO_4_] or Na_2_[WO_4_] in any mixing ratios produces mixtures of several types of (C_6_H_6_)Ru-POM complexes, as confirmed by the multiple singlets of POM-coordinated {Ru(C_6_H_6_)}^2+^ fragments between 5.8 and 5.5 ppm in their ^1^H NMR spectra (Figure S3). Note that we describe the composition of the reaction mixtures as the ratio between the *total* amount of Ru and Mo (or W); for example, a 1:1 Ru:Mo mixture contains 1 part of [{Ru(arene)Cl}_2_(μ-Cl)_2_] and 2 parts of Na_2_[MoO_4_]. In both Ru–Mo and Ru–W systems, the ^1^H NMR spectrum of the 1:1 mixture differs from that of mixtures having a higher mole fraction of [MoO_4_]^2–^ or [WO_4_]^2–^ (1:2, 1:3, or 1:4), indicating that the molar ratio of the reactants is one of the factors affecting the speciation of (C_6_H_6_)Ru-POM complexes. As shown in Scheme 2, we successfully isolated compounds **1**–**7** from the reaction mixtures by adjusting the crystallization temperature and Ru/Mo or Ru/W ratio. An exception is the synthesis of **7** in which crystallization of the 1:2 mixture of [{Ru(C_6_H_6_)Cl}_2_{μ-Cl}_2_] and Na_2_[WO_4_] at room temperature always produced a mixture of **6** and **7**. Each compound has a unique metal–oxygen framework structure, and they are discernible by their characteristic peaks between 950 and 450 cm^–1^ in the FTIR spectra (Figure S4). Moreover, their PXRD patterns (Figure S5) match with the simulated patterns using a single-crystal structure, confirming the bulk purity. Although the ^1^H NMR spectra of **1**–**3** show impurity peaks most likely due to decomposition of the solid upon dissolution, the ^1^H NMR spectra of **4**–**6** are in good agreement with the symmetry of their respective solid-state structure (Figures S6 and S7). Equations 2–8c express the formation of compounds **1**–**7**:
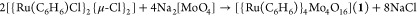
2
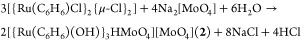
3
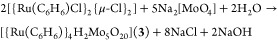
4
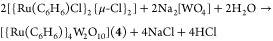
5
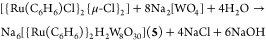
6
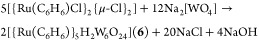
7
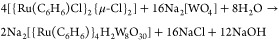
8a
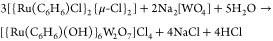
8b
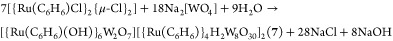
8c

### Crystal Structures of (C_6_H_6_)Ru–Polyoxomolybdate Species

**Figure 1:**
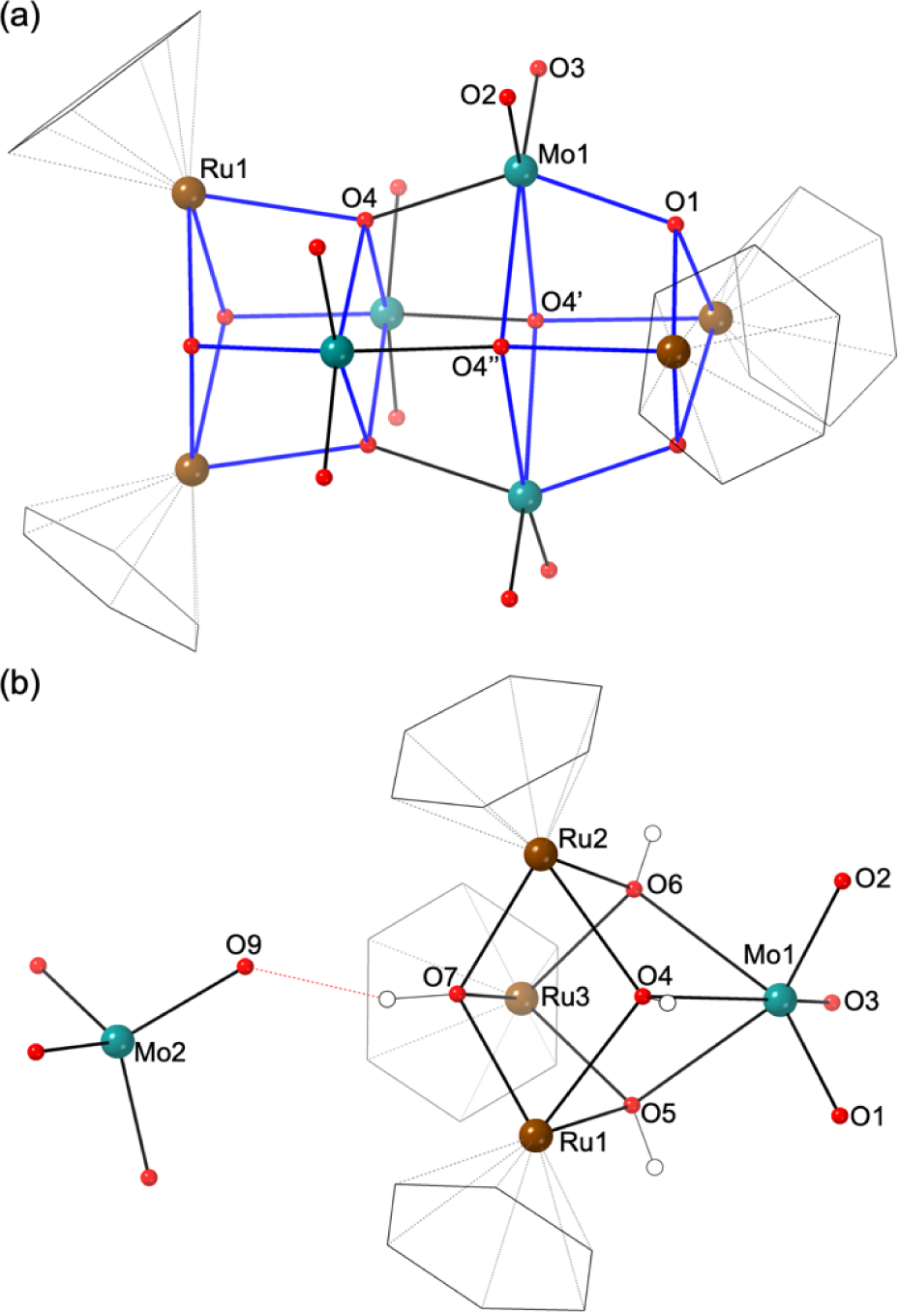
Structure of (a) **1** and (b) **2**. The cube-like {Ru_2_Mo_2_O_4_} building blocks in **1** are highlighted in blue lines. The dashed red line in **2** represents a hydrogen bond. Color scheme: teal, Mo; brown, Ru; red, O. Benzene ligands are drawn as a black wireframe.

Compound **1** crystallized as dark-brown crystals from a mixture of [{Ru(C_6_H_6_)Cl}_2_(μ-Cl)_2_] and Na_2_[MoO_4_] in a 1:5 Ru:Mo molar ratio at 70 °C, whereas **2** crystallized as yellow crystals from a concentrated 1:5 Ru:Mo mixture at 27 °C. As shown in Figure 1a, **1** adopts the so-called triple-cubane structure in which a cubane-like [Mo_4_O_16_]^8–^ anion is capped by four {Ru(C_6_H_6_)}^2+^ cations. Alternatively, we viewed the triple-cubane structure of **1** as being composed of two {Ru_2_Mo_2_O_4_} cubes, which are also the building block in **3**; a similar {Ru_2_W_2_O_4_} unit is also observed in **4** and **6**. There are three types of Mo–O distances: (i) short, terminal Mo–O bonds (Mo1–O2 and Mo1–O3, *d* = 1.71 Å), (ii) medium length, Mo1–O1 (*d* = 1.92 Å) and Mo1–O4 (*d* = 1.94 Å) bonds that make the edges of the triple-cubane framework, and (iii) long Mo1–O4′ (*d* = 2.36 Å) and Mo1–O4″ (*d* = 2.33 Å), which are *trans* to the terminal Mo1–O2 and Mo1–O3, respectively. The average Ru–O bond length is 2.1 Å. All bond parameters in **1** agree with the reported values of other [{Ru(arene)}_4_Mo_4_O_16_] complexes. Compounds having the formula [{ML}_4_M_4_O_16_] (where {ML} is metal–organic fragments) are common in organometallic-POM chemistry, and they could adopt triple-cubane or windmill-shaped structures depending on the types of the ligands and reaction conditions such as solvents.^21^ For example, [{Ru(*p*-cymene)}_4_Mo_4_O_16_] is isolated as the windmill isomer,^25^ whereas [{Ru(arene)}_4_Mo_4_O_16_] (arene = C_6_H_5_Me, 1,3,5-C_6_H_3_Me_3_, or 1,2,4,5-C_6_H_2_Me_4_) are isolated as the triple-cubane isomer.^19^ The Proust group has reported extensive study of the isomerization of [{Ru(arene)}_4_Mo_4_O_16_] clusters, and **1** provides the first crystal structural evidence that [{Ru(C_6_H_6_)}_4_Mo_4_O_16_] prefers a triple-cubane structure.

Compound **2** consists of a *cationic* (C_6_H_6_)Ru–molybdate complex, [{Ru(C_6_H_6_)(OH)}_3_HMoO_4_]^2+^, charge-balanced by a mononuclear [MoO_4_]^2–^ anion. The structure of the cationic part contains a {Ru_3_MoO_4_} single cubane featuring a tripod-like arm of a [{Ru(C_6_H_6_)(OH)}_3_]^3+^ trimer that catches a monoprotonated [HMoO_4_]^−^ anion (Figure 1b). The Mo–O distances and the BVS values (Table S2) indicated the three facial oxygen atoms (O1–O3, *d* Mo1–O1/O2/O3 = 1.73–1.75 Å) of the [HMoO_4_]^−^ unit are terminal oxo ligands, whereas the bridging oxygen atoms (O4–O7, *d* Mo1–O4/O5/O6 = 2.22–2.27 Å; mean *d* Ru–O7 = 2.10 Å) of the {Ru_3_MoO_4_} framework are monoprotonated (i.e., μ_3_-OH groups). In polyoxometalate chemistry, an octahedron with three terminal M=O bonds is uncommon because it violates Lipscomb’s restriction.^43^ However, the *fac*-{MoO_3_} could be stabilized by coordination with organic ligands such as tacn or Cp*.^44,45^ In the crystal packing, the cationic [{Ru(C_6_H_6_)(OH)}_3_HMoO_4_]^2+^ complex interacts with a tetrahedral [MoO_4_]^2–^ anion through a short OH···OMo hydrogen bond (*d* O7···O9 = 2.51 Å).

**Figure 2:**
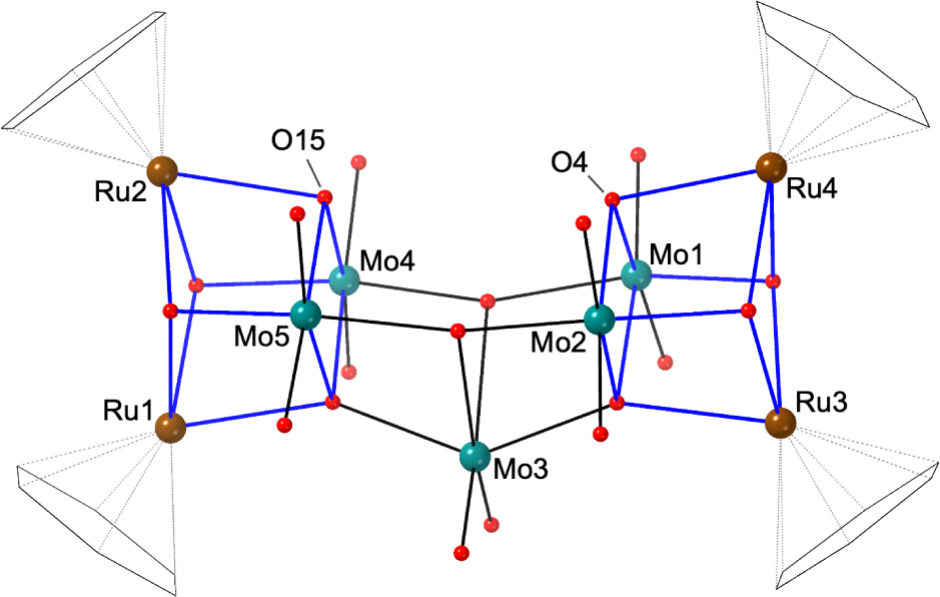
Structure of compound **3**. The cube-like {Ru_2_Mo_2_O_4_} building blocks are highlighted in blue lines. Color scheme: teal, Mo; brown, Ru; red, O. Benzene ligands are drawn as a black wireframe.

Crystallization of a mixture of [{Ru(C_6_H_6_)Cl}_2_(μ-Cl)_2_] and Na_2_[MoO_4_] in a 1:1 Ru:Mo ratio at 70 °C gave small, deep brown crystals of **3**. Compound **3** is a charge-neutral cluster composed of pentamolybdate anion [H_2_Mo_5_O_20_]^8–^ coordinated by four {Ru(C_6_H_6_)}^2+^ cations (Figure 2). The structure of the pentamolybdate anion is new and is relevant to the {Mo_6_O_22_} fragment in the [{RhCp*}_4_Mo_6_O_22_] tetra-cubane cluster^46^ by removing one of the central *cis*-{MoO_2_}^2+^. As such, there is a lacunary or defect site in **3**,^47^ and BVS calculations indicated the two oxygen atoms near the defect sites (O4, BVS = 1.14 and O15, BVS = 1.10) are monoprotonated. Note the chemical formula of the pentamolybdate [H_2_Mo_5_O_20_]^8–^ anion in **3** and the tetramolybdate [Mo_4_O_16_]^8–^ anion in **1** can be written as an aggregate of monomeric molybdate anions [(HMoO_4_)_2_(MoO_4_)_3_]^8–^ and [(MoO_4_)_4_]^8–^, suggesting that their formation may not involve acidic condensation reactions but direct coordination of molybdate anions to the ruthenium centers.

### Crystal Structures of (C_6_H_6_)Ru–Polyoxotungstate Species

**Figure 3:**
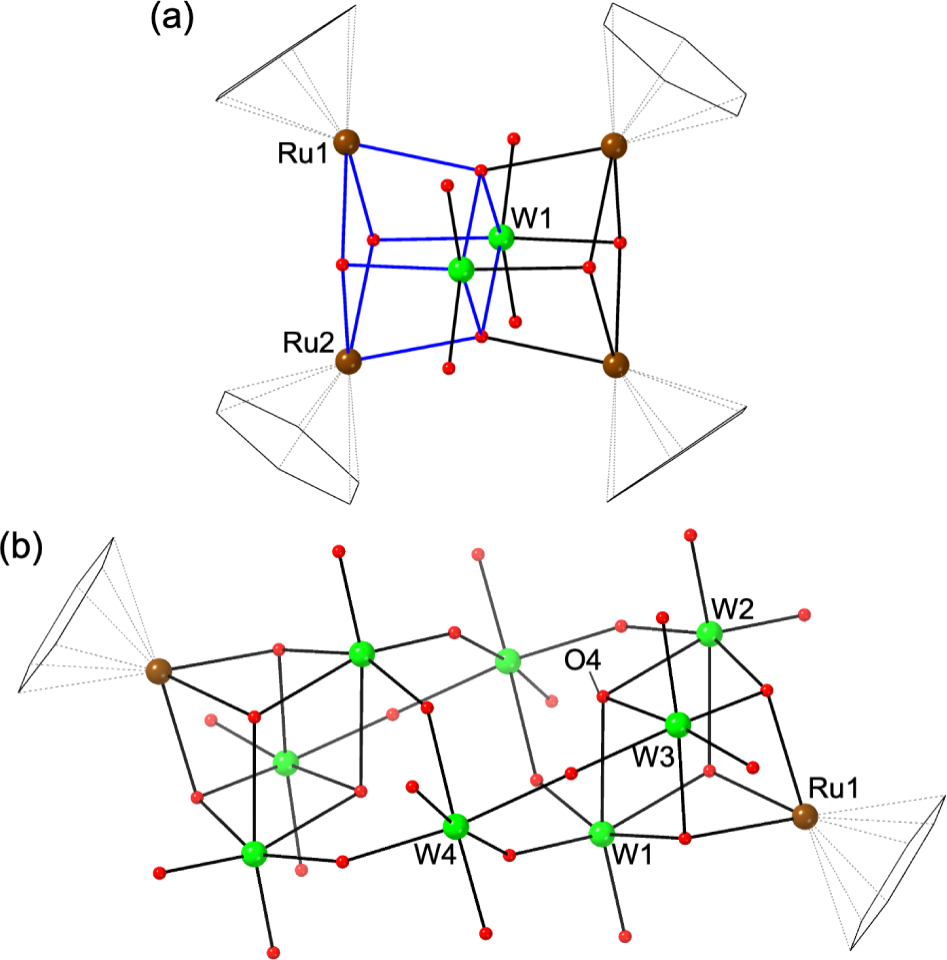
Structure of (a) **4** and (b) the anionic part of **5**. The blue lines in **4** emphasize the {Ru_2_W_2_O_4_} unit. Color scheme: green, W; brown, Ru; red, O. Benzene ligands are drawn as a black wireframe.

Compounds **4** and **5** were isolated from the mixture of [{Ru(C_6_H_6_)Cl}_2_(μ-Cl)_2_] and Na_2_[WO_4_] in a 1:5 Ru:W molar ratio by fractional crystallization at high and low temperatures: **4** crystallized first at 80 °C, whereas crystals of **5** grew from the remaining mother liquor at room temperature. The two compounds are distinguishable by their crystal appearance: well-developed reddish-brown blocks for **4** and yellow rectangular blocks for **5**. Compound **4** is a charge-neutral cluster comprising an edge-sharing ditungstate [W_2_O_10_]^8–^ anion capped by four {Ru(C_6_H_6_)}^2+^ cations, forming a double cubane structure (Figure 3a). BVS calculations showed that all oxygen atoms are oxo ligands. The structure is similar to that of [{Ru(*p*-cymene)}_4_W_2_O_10_] produced as a byproduct from the reaction of [{Ru(*p*-cymene)Cl}_2_(μ-Cl)_2_] and [N(C_4_H_9_)_4_]_2_[WO_4_] in organic media.^26^

Compound **5** is a sodium salt of an anionic [{Ru(C_6_H_6_)}_2_H_2_W_8_O_30_]^6–^, which has been reported by the Bi group^29^ and the Abramov group.^30^ As shown in Figure 3b, the anionic part contains an octatungstate [H_2_W_8_O_30_]^10–^ cluster, which is capped by two {Ru(C_6_H_6_)}^2+^ units. The structure is considered as two cubane-shaped {Ru(C_6_H_6_)(HW_3_O_13_)}^5–^ fragments linked by two *cis*-{WO_2_}^2+^ cations, similar to the structure of [{Ir(C_5_Me_5_)}_2_H_2_W_8_O_30_]^6–^ reported by us.^35^ BVS calculations confirmed that O4 is the protonation site (BVS = 1.23). In the crystal packing, the anion is connected to one another by sodium cations (Figure S8).

**Figure 4:**
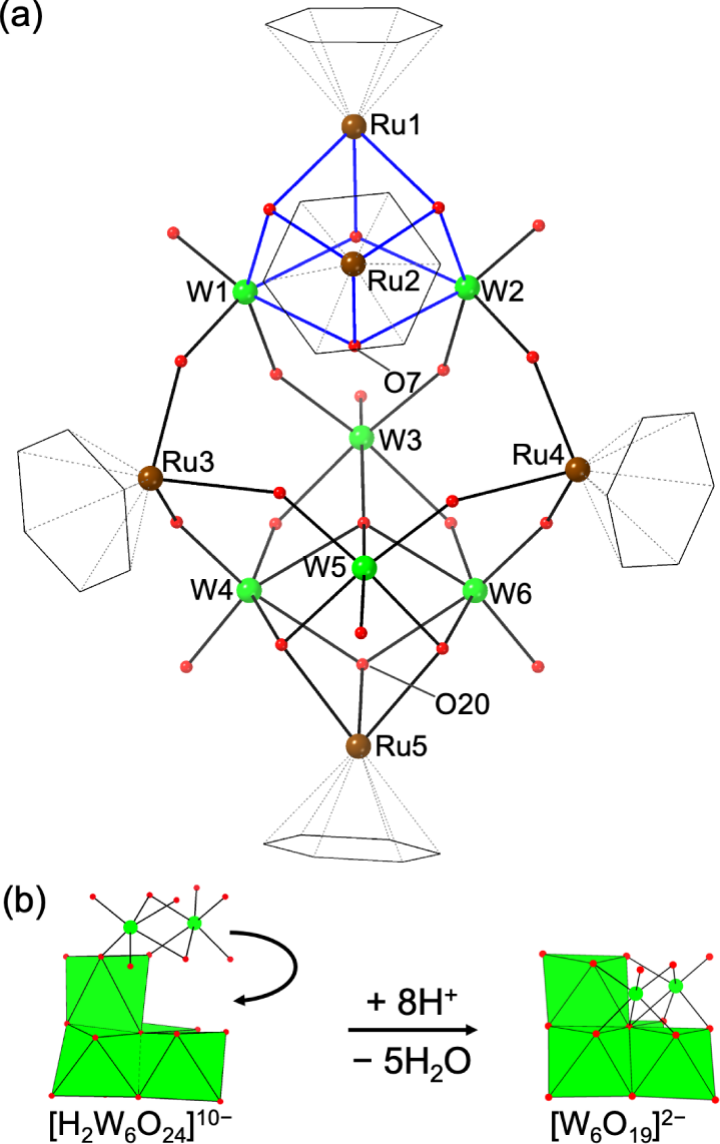
(a) Structure of **6** and (b) reaction scheme showing a hypothetical transformation of the hexatungstate core in **6** into the Lindqvist anion. Color scheme: green, W; brown, Ru; red, O. Benzene ligands are drawn as a black wireframe.

Crystallization of a mixture of [{Ru(C_6_H_6_)Cl}_2_(μ-Cl)_2_] and Na_2_[WO_4_] in a 1:1 Ru:W ratio at 70 °C produces brown crystals of **6**, whereas crystallization of the same mixture at 27 °C produces a mixture of brown crystals of **6** and needlelike yellow crystals of **7**. We obtained crystals of **7** suitable for X-ray analysis by adding 0.1 M sodium acetate to the reaction mixture. X-ray diffraction showed that crystals grown with and without sodium acetate had the same unit cell and hence were the same compound. Compound **6** is composed of a new hexatungstate [H_2_W_6_O_24_]^10–^ anion and five {Ru(C_6_H_6_)}^2+^ cations that coordinate to one side of the anion, forming a molecule having hydrophobic surfaces on one side and hydrophilic surfaces on the other sides (Figure 4a; a space-filling model is given in Figure S9). The hexatungstate [H_2_W_6_O_24_]^10–^ anion can be viewed as an “open” form of a Lindqvist [W_6_O_19_]^2–^ anion, and Figure 4b shows a hypothetical transformation of [H_2_W_6_O_24_]^10–^ to [W_6_O_19_]^2–^. BVS calculations (Table S2) indicated that sites O7 (BVS, 1.19) and O20 (BVS, 1.31) are the protonation sites. Both **6** and **4** contain a {Ru_2_W_2_O_4_} single-cubane building block: {Ru_2_W_2_O_4_} in **4** is protected by the coordination of two {Ru(C_6_H_6_)}^2+^ cations, whereas in **6**, it links to a tetratungstate fragment and forms the hexatungstate anion.

**Figure 5:**
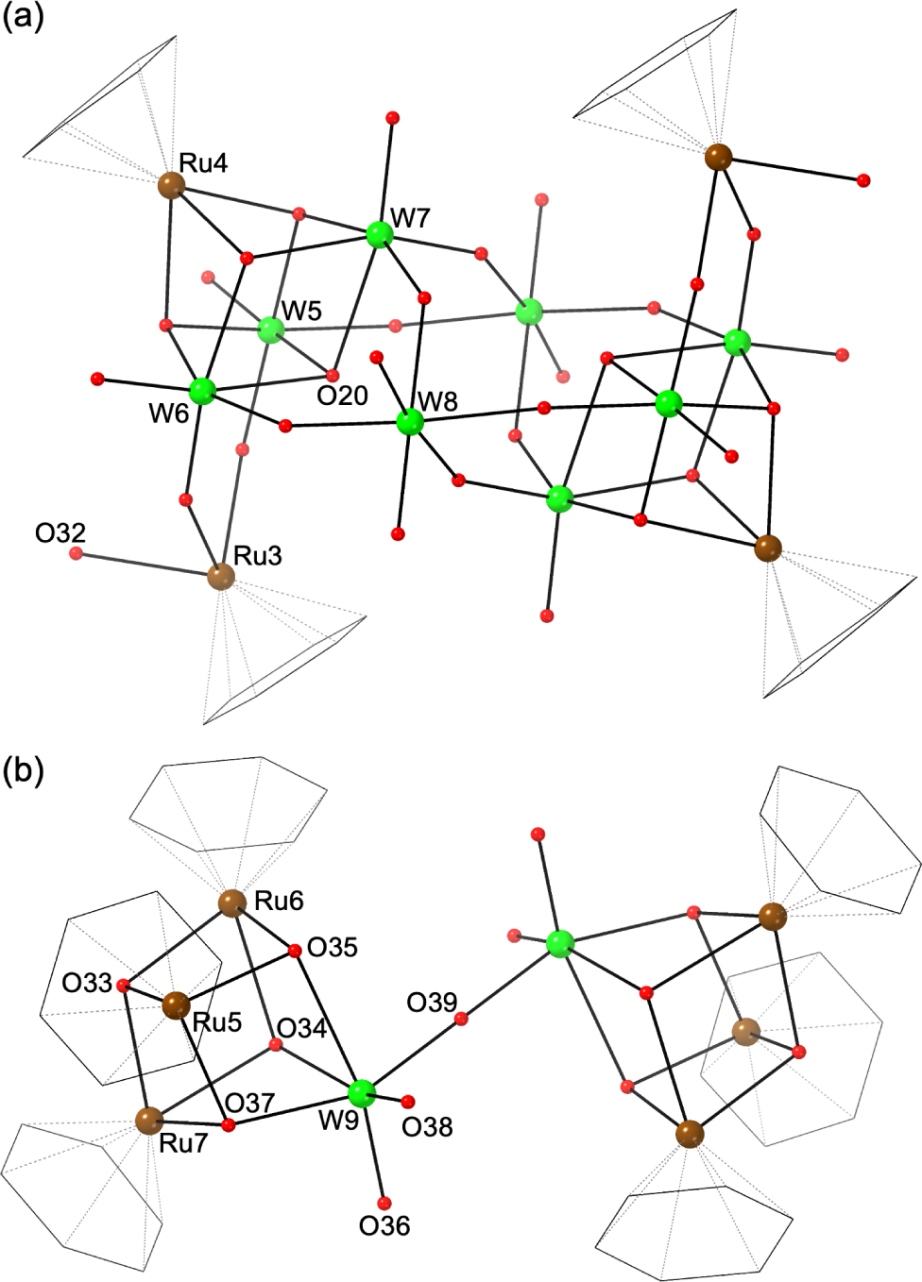
Structure of (a) the anionic part and (b) the cationic part of **7**. Color scheme: green, W; brown, Ru; red, O. Benzene ligands are drawn as a black wireframe. O32 is a water molecule coordinating Ru3.

Compound **7** comprises a new cationic cluster [{Ru(C_6_H_6_)(OH)}_6_W_2_O_7_]^4+^ charge-balanced by two anionic [{Ru(C_6_H_6_)}_2_{Ru(C_6_H_6_)(OH_2_)}_2_H_2_W_8_O_30_]^2–^ clusters. The structure of the anion is similar to that of [{Ru(*p*-cymene)}_2_{Ru(*p*-cymene)(OH_2_)}_2_H_2_W_8_O_30_]^2–^ reported by Proust et al. (Figure 5a).^26^ Note that the same anionic structure is mentioned by Bi et al., but the structure is not reported.^29^ The cationic cluster [{Ru(C_6_H_6_)(OH)}_6_W_2_O_7_]^4+^ consists of a corner-sharing {W_2_O_7_}^2–^ dimer capped by two {Ru(C_6_H_6_)(OH)}_3_ trimers, forming a structure having two {Ru_3_WO_4_} cubes connected by a bridging oxygen atom (Figure 5b). BVS values of O33 (1.2), O34 (1.30), and O35 (1.19) indicate they are hydroxo groups. In the crystal packing, the O33 forms hydrogen bonds to a terminal oxo ligand (O6) of the neighboring anionic clusters, forming chain-like cation–anion pairs propagating along the [111] direction (Figure S10). As such, the metal–oxygen backbone of [{Ru(C_6_H_6_)(OH)}_6_W_2_O_7_]^4+^ could be viewed as the dimer of the cationic part of **2**, and the dimerization reaction—ignoring the type of the metal centers—is shown in eq 9.
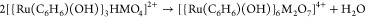
9

### Thermal Stability of the Hydrogen-Bonded Framework of **1**

**Figure 6:**
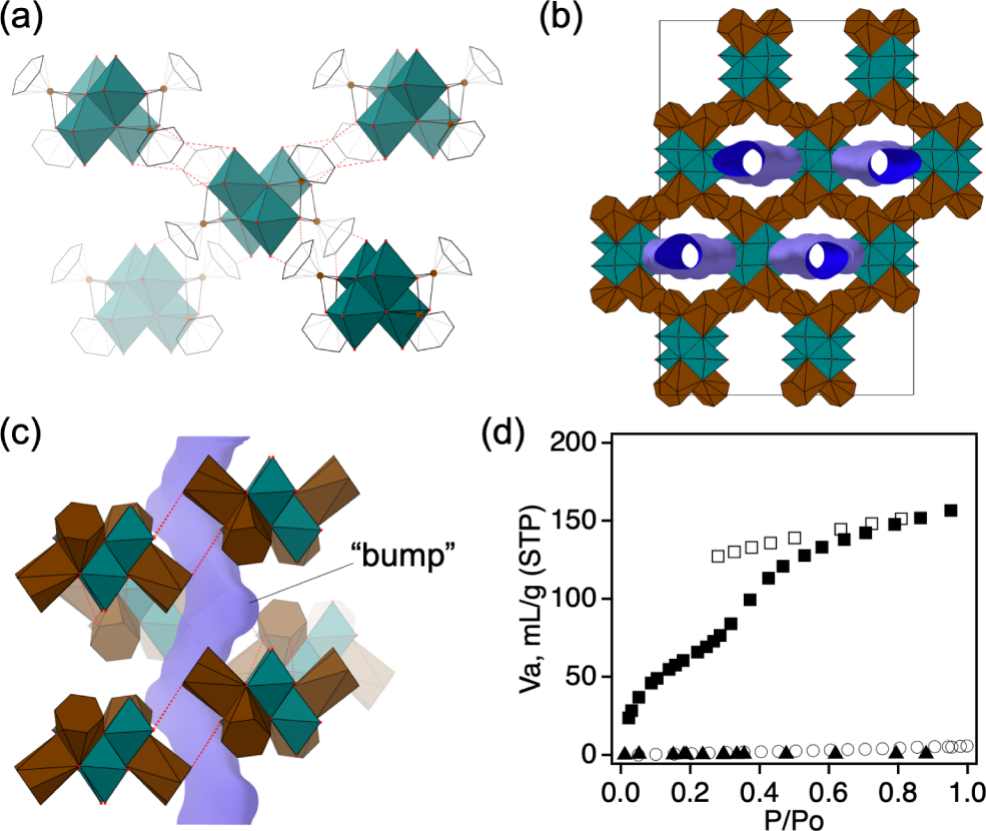
(a) Intermolecular C–H···O hydrogen bonds (red dotted lines) in **1**, (b) crystal packing of **1** projected along the *a* direction with water channels shown in blue, (c) a view of the channel showing its curved shape and bumps, and (d) water-vapor adsorption (filled squares) and desorption (open squares) isotherm at 25 °C, nitrogen adsorption isotherm (filled triangles) at −196 °C, and carbon dioxide adsorption isotherm (open circles) at 25 °C.

Compound **1** comprises charge-neutral [{Ru(C_6_H_6_)}_4_Mo_4_O_16_] molecules. Each [{Ru(C_6_H_6_)}_4_Mo_4_O_16_] interacts with four neighboring molecules through nonconventional C–H···O hydrogen bonds with an average C···O distance of 3.4 Å (Figure 6a), forming three-dimensional hydrogen-bonded frameworks in the crystal structure and channels along *a* direction (Figure 6b). The channels are curved and have bumps or swollen areas (Figure 6c) with an aperture dimension of approximately 4.5 × 2.8 Å^2^, and they host water solvates. The crystal of **1** loses its long-range order upon dehydration at 100 °C but regains its crystallinity by absorbing water vapor from the air. PXRD patterns of **1** dehydrated by (i) heating in a heating device under a nitrogen flow (Figure S11) and (ii) after dehydration at 100 °C under a vacuum condition (Figure S12) showed only broad and weak peaks. When the dehydrated solid was exposed to the air, it again showed the characteristic intense and sharp PXRD profile of **1**. As such, these PXRD pattern changes confirm that **1** undergoes reversible crystal-to-amorphous-to-crystal phase transitions upon dehydration–rehydration cycles. Furthermore, TG analyses (Figure S13) showed that 90% and 96% of the sample were rehydrated after exposure to ambient air for 5 and 20 min, respectively. Although dehydrated **1** showed additional peaks in the FTIR spectrum, its ^1^H NMR spectrum is similar to that of the pristine sample (Figure S14), suggesting that the structure of [{Ru(C_6_H_6_)}_4_Mo_4_O_16_] is maintained after the phase transformation. The water-vapor sorption isotherm (Figure 6d) showed that the maximum volume of water adsorbed was 156.6 mL (STP) per gram of solid, corresponding to 9.5 mol of water solvates per mol of [{Ru(C_6_H_6_)}_4_Mo_4_O_16_]. This value is close to the number of hydrates found by elemental and thermogravimetric analyses. On the other hand, nitrogen and carbon dioxide gas did not enter the pores of the dehydrated **1**, due to different kinetic diameters (N_2_, 3.64 Å; CO_2_, 3.30 Å; H_2_O, 2.65 Å) and/or different abilities to make hydrogen bonding. The two-step water-vapor adsorption process might be due to (i) the adsorption of water into the channel’s bumps or (ii) the recovery of a long-range order of the hydrogen-bonded network.

## Conclusion

The reaction of [{Ru(C_6_H_6_)Cl}_2_(μ-Cl)_2_] and Na_2_[MoO_4_] or Na_2_[WO_4_] produces several kinds of (C_6_H_6_)Ru-grafted POM species, and seven compounds are isolable by adjusting the molar ratio of the reactants and crystallization temperature. {Ru(C_6_H_6_)}^2+^ cations stabilize small and reactive POM building blocks and guide their linkage into new, cubane-like metal–oxygen backbones. The molecular crystal of [{Ru(C_6_H_6_)}_4_Mo_4_O_16_] contains extensive intramolecular C–H···O hydrogen bonds, and it undergoes a reversible crystal-to-amorphous-to-crystal transformation upon dehydration–rehydration cycles. As such, this work demonstrates that organometallic cations are versatile structure-directing agents for POM syntheses, and the resulting organometallic oxide clusters provide opportunities to explore flexible porous crystals based on purely noncovalent interactions such as hydrogen bonds and pi interactions.

## Supporting Information Available

Additional experimental results (PXRD patterns, thermograms, FTIR spectra, and ^1^H NMR spectra), crystallographic parameters, and BVS calculation results (PDF)
