# Supplementary material for: Directing the Linkage of Small Polyoxometalate Building Blocks Using (Benzene)ruthenium Cations
Source: Inorg Chem. 2024 Dec 10;63(51):24240–9. doi: 10.1021/acs.inorgchem.4c04150 (PMC11684009; doi:10.1021/acs.inorgchem.4c04150)
Supplement: Supplementary file 1 — ic4c04150_si_001.pdf [file ic4c04150_si_001.pdf]

Supporting Information

Directing the linkage of small polyoxometalate building blocks using (benzene)ruthenium cations

Sugiarto,[a] Yuudai Iwai,[b] Ryo Ohtani,[b] and Masahiro Sadakane,[a]*

[a]Department of Applied Chemistry, Graduate School of Advanced Science and Engineering, Hiroshima University, 1-4-1 Kagamiyama, Higashi-Hiroshima 739-8527, Japan

[b]Department of Chemistry, Faculty of Science, Kyushu University, 744 Motooka, Nishi-ku, Fukuoka 819-0395, Japan

*Corresponding author: sadakane09@hiroshima-u.ac.jp

Contents

Table S1. Crystallographic parameters S2

Table S2. Bond valence sum (BVS) of Mo, W, and O atoms S4

Figure S1. Thermograms of **1**–**3** S6

Figure S2. Thermograms of **4**–**7** S7

Figure S3. ^{1}H NMR spectra of reaction mixtures S8

Figure S4. FTIR spectra of **1**–**7** S9

Figure S5. PXRD patterns of **1**–**7** S10

Figure S6. ^{1}H NMR spectra of **1**–**3** S11

Figure S7. ^{1}H NMR spectra of **4**–**6** S12

Figure S8. Crystal packing of **5** S13

Figure S9. Space filling model of **6** S14

Figure S10. Cation···anion pairs in **7** S15

Figure S11. PXRD patterns of **1** under heating in a heating device S16

Figure S12. PXRD pattern of **1** dehydrated at 100°C under vacuum S17

Figure S12. Thermograms of **1** after dehydration and re-hydration in the air S18

Figure S13. FTIR and ^{1}H NMR spectra of **1** before and after dehydration S19

Table S1. Crystallographic parameters. * Note that formulas and formula weights are based on X-ray diffraction results.

	**1 (Ru4Mo4)**	**2 (Ru3Mo)**	**3 (Ru4Mo5)**
Formula unit*	$C_{24}H_{24}Mo_4O_{16}Ru_4$	$C_{18}H_{22}Mo_2O_{18}Ru_3$	$C_{24}H_{24}Mo_5O_{24}Ru_4$
Formula weight* (g/mol)	1356.47	1021.44	1580.41
Crystal system	Orthorhombic	Orthorhombic	Monoclinic
Space group (number)	*Fddd* (70)	$P2_12_12_1$ (19)	$P2_1/c$ (14)
a, Å	11.0002(2)	11.2328(1)	17.6099(3)
b, Å	21.4859(3)	12.9594(2)	12.2494(2)
c, Å	31.9746(4)	20.1868(2)	17.7412(3)
α, °	90	90	90
β, °	90	90	91.086(2)
γ, °	90	90	90
V, Å^3	7557.2(2)	2938.60(6)	3826.28(11)
Z	8	4	4
D_{calc} (g/cm^3)	2.384	2.309	2.743
μ (mm^{-1})	2.897	2.413	3.202
Radiation	Mo Kα (λ = 0.71073 Å)	Mo Kα (λ = 0.71073 Å)	Mo Kα (λ = 0.71073 Å)
Temperature (K)	123(2)	123(2)	123(2)
F_{000}	5120	1960	2984
Θ range	2.175 to 27.480°	1.867 to 25.242°	2.020 to 26.372°
Index ranges	$-14 \le h \le 14$<br>$-27 \le k \le 27$<br>$-41 \le l \le 41$	$-14 \le h \le 14$<br>$-16 \le k \le 16$<br>$-23 \le l \le 26$	$-21 \le h \le 22$<br>$-15 \le k \le 15$<br>$-22 \le l \le 22$
No. of reflections collected	23556	41020	42670
Unique reflections (R_{int})	2173 (0.0279)	6745 (0.0373)	7813 (0.0383)
Data/restrains/parameters	2173/73/110	6745/4/382	7813/528/569
R indexes [I>2σ(I)]	$R_1^a = 0.0224$<br>$wR_2^b = 0.0479$	$R_1 = 0.0180$<br>$wR_2 = 0.0461$	$R_1 = 0.0315$<br>$wR_2 = 0.0773$
R indexes (all data)	$R_1 = 0.0227$<br>$wR_2 = 0.0480$	$R_1 = 0.0185$<br>$wR_2 = 0.0463$	$R_1 = 0.0385$<br>$wR_2 = 0.0801$
Goodness-of-fit	1.349	1.045	1.033
$(\Delta/\sigma)_{max}$	0.001	0.002	0.001
$\Delta\rho_{max/min}$ (e A^{-3})	0.482/−0.620	0.645/−0.599	5.523/−1.357
CCDC number	2349200	2349201	2349202

$^aR_1 = \{\Sigma||F_o|-|F_c||\}/\{\Sigma|F_o|\}$; $^bwR_2 = [\Sigma w(F_0^2-F_c^2)/\Sigma wF_o^2]^{1/2}$

Table S1 (*continued from page S2*)

	**4 (Ru4W2)**	**5 (Ru2W8)**	**6 (Ru5W6)**	**7 (Ru6W2)**
Formula unit*	$C_{24}H_{24}O_{20}Ru_4W_2$	$C_{12}H_{12}Na_{5.8}O_{46.3}Ru_2W_8$	$C_{30}H_{30}O_{41.85}Ru_5W_6$	$C_{84}H_{84}O_{111}Ru_{14}W_{18}$
Formula weight* (g/mol)	1404.41	2703.3	2668.59	7593.79
Crystal system	Triclinic	Monoclinic	Monoclinic	Triclinic
Space group (number)	$P\bar{1}$ (2)	$P2_1/n$ (14)	$P2_1/c$ (14)	$P\bar{1}$ (2)
a, Å	8.8788(1)	10.3610(4)	16.2903(14)	12.0806(2)
b, Å	9.0131(1)	16.5542(5)	20.7364(18)	21.0050(6)
c, Å	22.6804(3)	15.1514(5)	19.1220(17)	21.9750(5)
α, °	79.789(1)	90	90	116.458(3)
β, °	85.151(1)	102.213(3)	97.310(1)	90.889(2)
γ, °	86.925(1)	90	90	95.222(2)
V, Å^3	1783.67(4)	2539.92(15)	6407.0(10)	4961.6(2)
Z	2	2	4	1
D_{calc} (g/cm^3)	2.615	3.535	2.767	2.541
μ (mm^{-1})	8.152	18.772	11.957	11.496
Radiation	Mo Kα (λ = 0.71073 Å)	Mo Kα (λ = 0.71073 Å)	Mo Kα (λ = 0.71073 Å)	Mo Kα (λ = 0.71073 Å)
Temperature (K)	123(2)	123(2)	123(2)	123(2)
F_{000}	1304	2396	4835	3424
Θ range	2.297 to 27.484°	1.845 to 25.350°	1.260 to 27.535°	1.948 to 25.350°
Index ranges	$-11 \leq h \leq 11$<br>$-11 \leq k \leq 11$<br>$-29 \leq l \leq 29$	$-12 \leq h \leq 12$<br>$-12 \leq k \leq 19$<br>$-18 \leq l \leq 17$	$-10 \leq h \leq 21$<br>$-26 \leq k \leq 26$<br>$-23 \leq l \leq 24$	$-14 \leq h \leq 14$<br>$-25 \leq k \leq 25$<br>$-26 \leq l \leq 26$
No. of reflections collected	27171	11987	35955	53570
Unique reflections (R_{int})	8186 (0.0337)	4628 (0.0258)	14509 (0.0405)	18170 (0.0770)
Data/restrains/parameters	8186/192/451	4628/104/356	14509/867/854	18170/825/931
R indexes [I>2σ(I)]	R_1 = 0.0251<br>wR_2 = 0.0657	R_1 = 0.0344<br>wR_2 = 0.0767	R_1 = 0.0480<br>wR_2 = 0.1040	R_1 = 0.0508<br>wR_2 = 0.1171
R indexes (all data)	R_1 = 0.0263<br>wR_2 = 0.0662	R_1 = 0.0373<br>wR_2 = 0.0778	R_1 = 0.0693<br>wR_2 = 0.1123	R_1 = 0.0746<br>wR_2 = 0.1257
Goodness-of-fit	1.141	1.140	1.080	1.011
$(\Delta/\sigma)_{max}$	0.002	0.001	0.012	0.001
$\Delta\rho_{max/min}$ (e A^{-3})	1.186/−1.901	1.619/−1.392	2.456/−1.841	5.138/−2.141
CCDC number	2349203	2349204	2349205	2349206

$^aR_1 = \{\Sigma||F_o|-|F_c||\}/\{\Sigma|F_o|\}$; $^bwR_2 = [\Sigma w(F_0^2-F_c^2)/\Sigma wF_o^2]^{1/2}$

Table S2. BVS calculation results showing the oxidation state of Mo or W atoms and the protonation state of O atoms. Mono-protonated oxygen atoms (OH) are shown in red and di-protonated oxygen atoms (OH_2) in blue.

**1**		**2**		**3**			
BVS of Mo atom(s)							
Mo1	5.89	Mo1	5.97	Mo1	6.00	Mo4	5.96
				Mo2	5.98	Mo5	5.99
				Mo3	5.91		
BVS of O atoms bind *solely* to Mo atoms							
O2	1.69	O1	1.63	O3	1.75	O12	1.74
O3	1.69	O2	1.53	O5	1.77	O13	1.77
		O3	1.60	O6	1.64	O14	1.66
				O7	1.70	O17	1.75
				O8	2.14	O18	1.70
				O10	2.12		
				O11	1.69		
BVS of O atoms bind *simultaneously* to Mo and Ru atoms							
O1	1.78	O4	1.22	O1	1.77	O16	1.95
O4	1.93	O5	1.21	O2	1.76	O19	1.79
		O6	1.18	O4	1.14	O20	1.80
		O7	1.2[a]	O9	1.92		
				O15	1.10		

Notes:

(a) The oxygen atom of a μ_3-OH group that bridges three Ru centers.

(b) The oxygen atom of a terminal aquo ligand on Ru.

Table S2 (*continued from page S3*)

4		5		6				7			
BVS of W atom(s)											
W1	6.02	W1	6.07	W1	6.08	W4	6.00	W1	6.03	W6	6.08
W2	5.96	W2	6.11	W2	6.07	W5	6.08	W2	6.02	W7	6.00
		W3	6.18	W3	6.07	W6	6.08	W3	6.06	W8	6.11
		W4	6.12					W4	5.76	W9	6.05
								W5	6.15		
BVS of O atoms bind *solely* to W atoms											
O1	1.57	O3	1.67	O1	1.67	O13	1.87	O3	2.06	O21	2.04
O2	1.60	O4	1.23	O3	1.68	O15	1.79	O4	1.23	O22	1.66
O6	1.58	O5	1.80	O9	2.09	O19	1.64	O5	2.07	O24	1.67
O7	1.59	O6	1.72	O10	2.07	O21	1.68	O6	1.60	O25	1.85
		O7	1.70	O11	1.73	O24	1.61	O8	1.66	O26	1.82
		O8	1.60	O12	1.94			O11	1.88	O29	1.58
		O9	1.99					O12	1.87	O30	1.66
		O11	1.66					O13	1.58	O31	1.61
		O12	1.67					O14	1.47	O36	1.70
		O13	1.63					O15	1.41	O38	1.57
		O14	1.58					O19	2.05	O39	2.13
		O15	2.09					O20	1.26		
BVS of O atoms bind *simultaneously* to Mo and Ru atoms											
O3	1.77	O1	1.74	O2	1.69	O17	1.74	O1	1.83	O23	1.82
O4	1.69	O2	1.74	O4	1.72	O18	1.87	O2	1.80	O27	1.85
O5	1.79	O10	1.86	O5	1.70	O20	1.31	O7	1.82	O28	1.88
O8	1.68			O6	1.90	O22	1.94	O9	1.77	O32	0.4[b]
O9	1.77			O7	1.19	O23	1.90	O10	1.82	O33	1.2[c]
O10	1.73			O8	1.85			O16	0.4[b]	O34	1.30
				O14	1.87			O17	1.77	O35	1.19
				O16	1.91			O18	1.82	O37	1.63

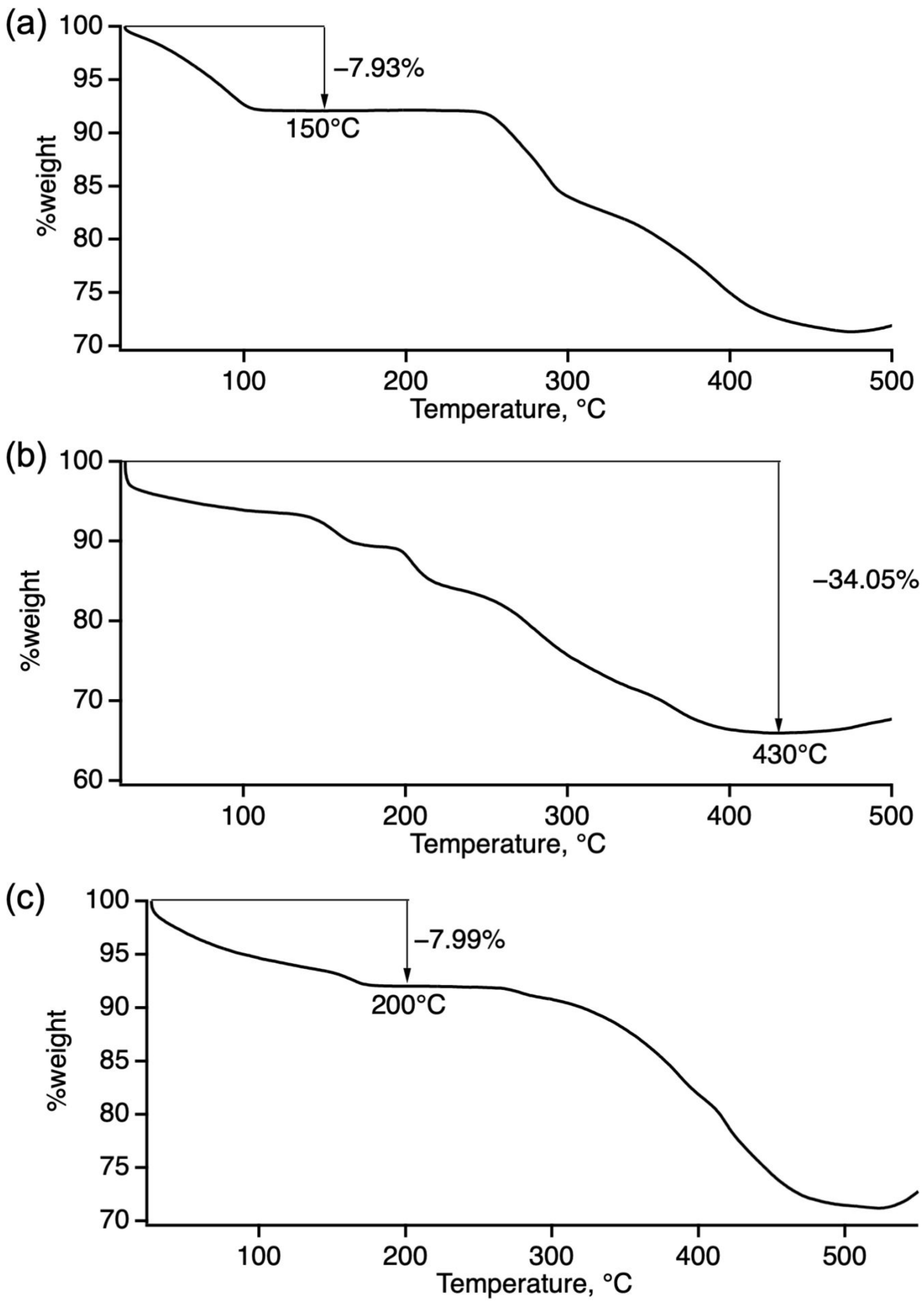

Figure S1. Thermograms of (a) **1**, (b) **2**, and (c) **3**.

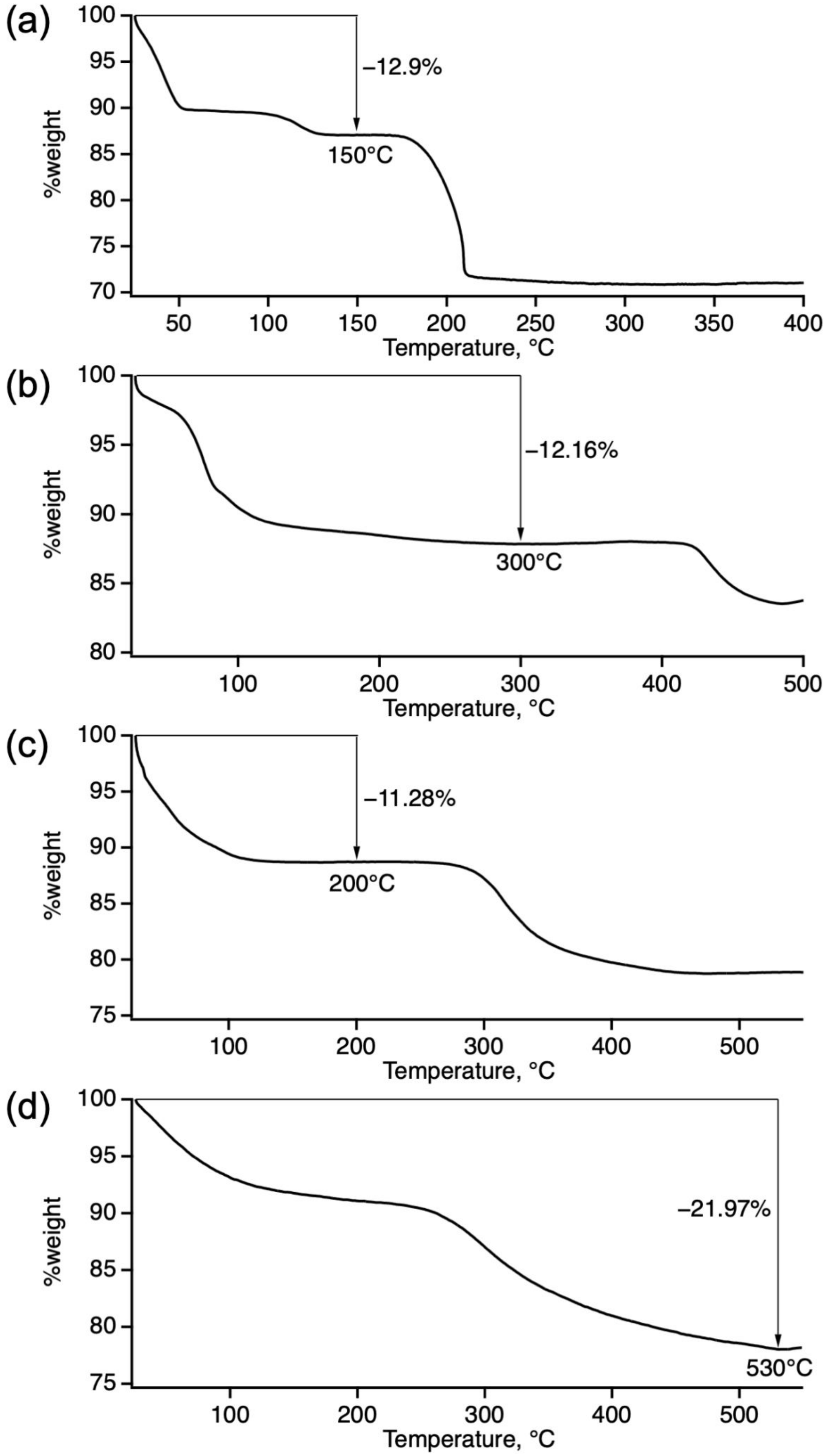

Figure S2. Thermograms of (a) **4**, (b) **5**, (c) **6**, and (d) **7**.

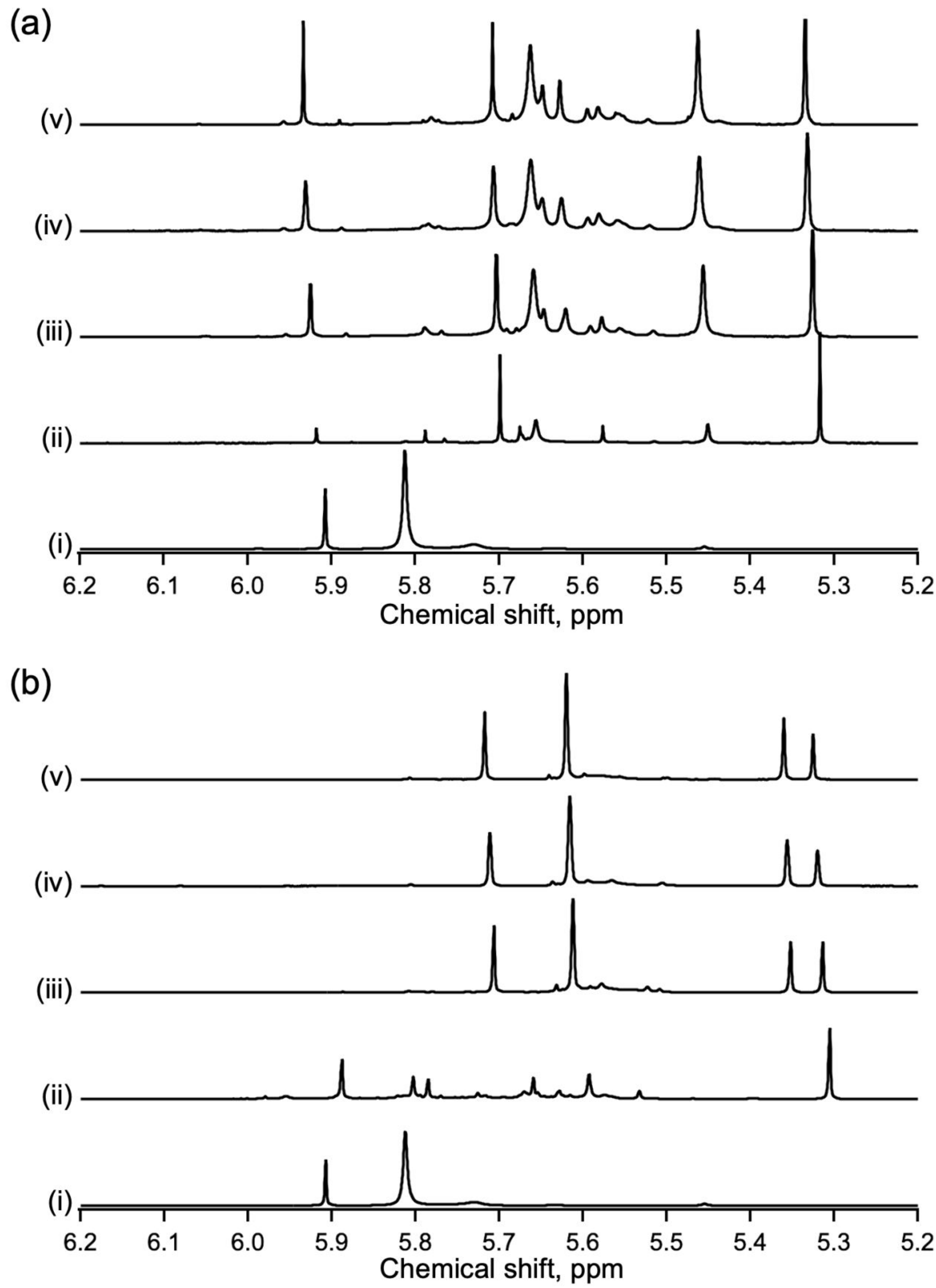

Figure S3. ^{1}H NMR spectra of the mixture of $[\{Ru(C_6H_6)Cl\}_2(\mu\text{-}Cl)_2]$ with (a) $Na_2[MoO_4]$ and (b) $Na_2[WO_4]$ in deuterium oxide in 1:0 (pure $[\{Ru(C_6H_6)Cl\}_2(\mu\text{-}Cl)_2]$, trace i), 1:1 (trace ii), 1:2 (trace iii), 1:3 (trace iv), and 1:4 (trace v) Ru:Mo or Ru:W molar ratios. The amount of Ru is approximately 6.2 mg (0.0125 mmol) and the volume of deuterium oxide was 0.7 mL.

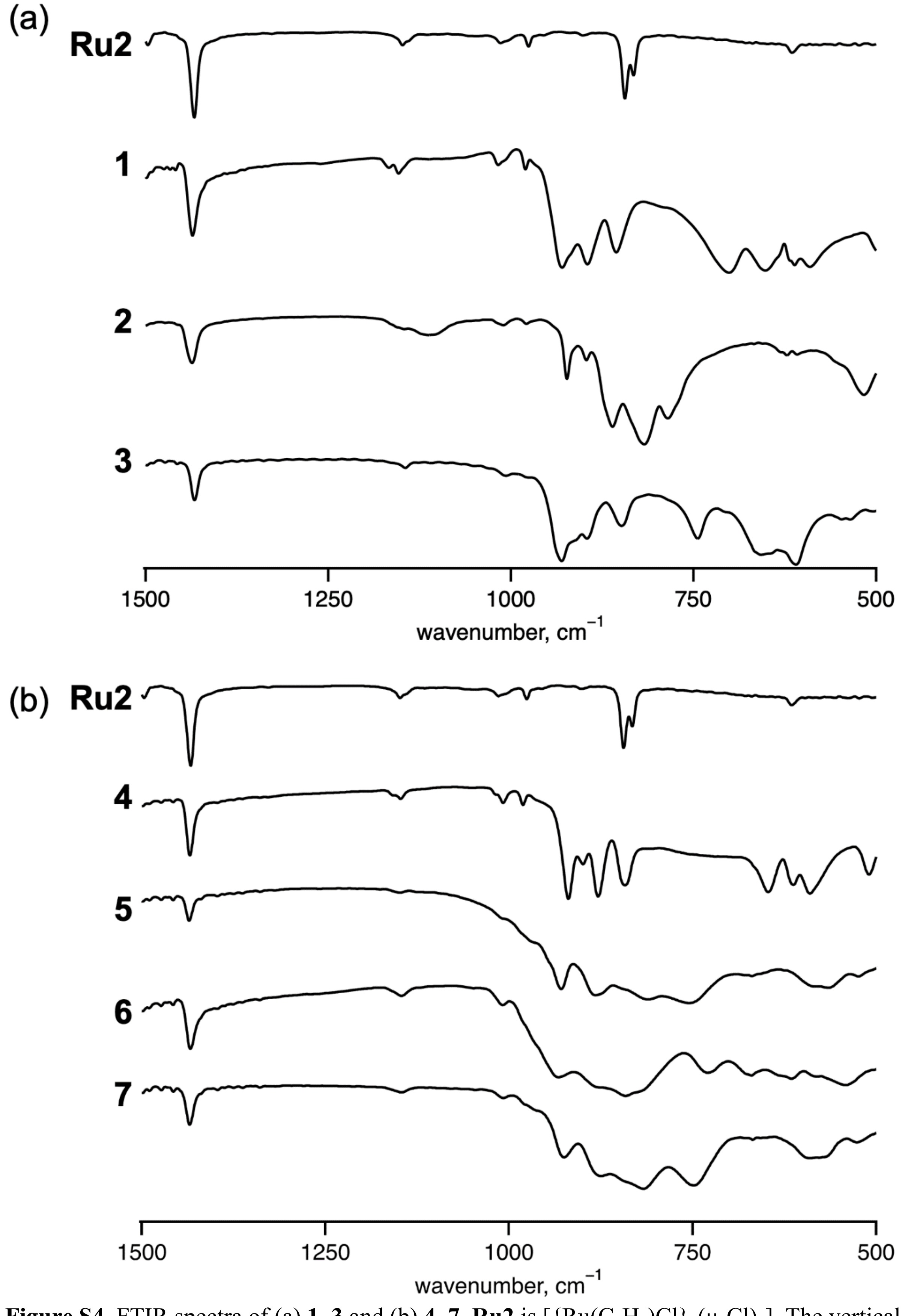

Figure S4. FTIR spectra of (a) **1**–**3** and (b) **4**–**7**. **Ru2** is [{Ru(C_6H_6)Cl}$_2$(μ-Cl)$_2$]. The vertical axis is %transmittance.

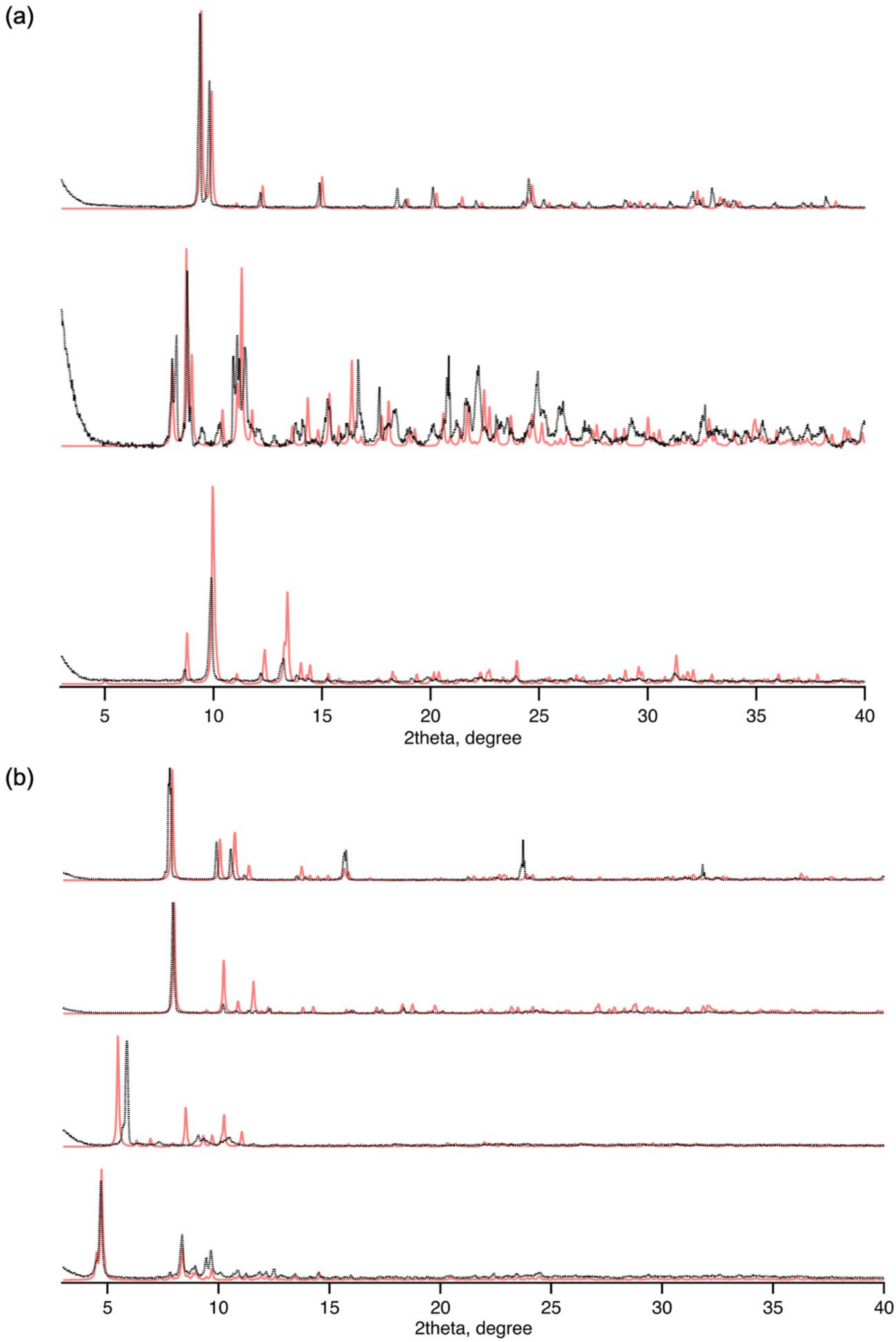

Figure S5. PXRD patterns (dotted black traces) of (a) **1**–**3** and (b) **4**–**7** in comparison with their respective simulated patterns based on single-crystal structure (translucent red traces). The mismatch between simulated and observed PXRD patterns of **6** is caused by the loss of hydrates.

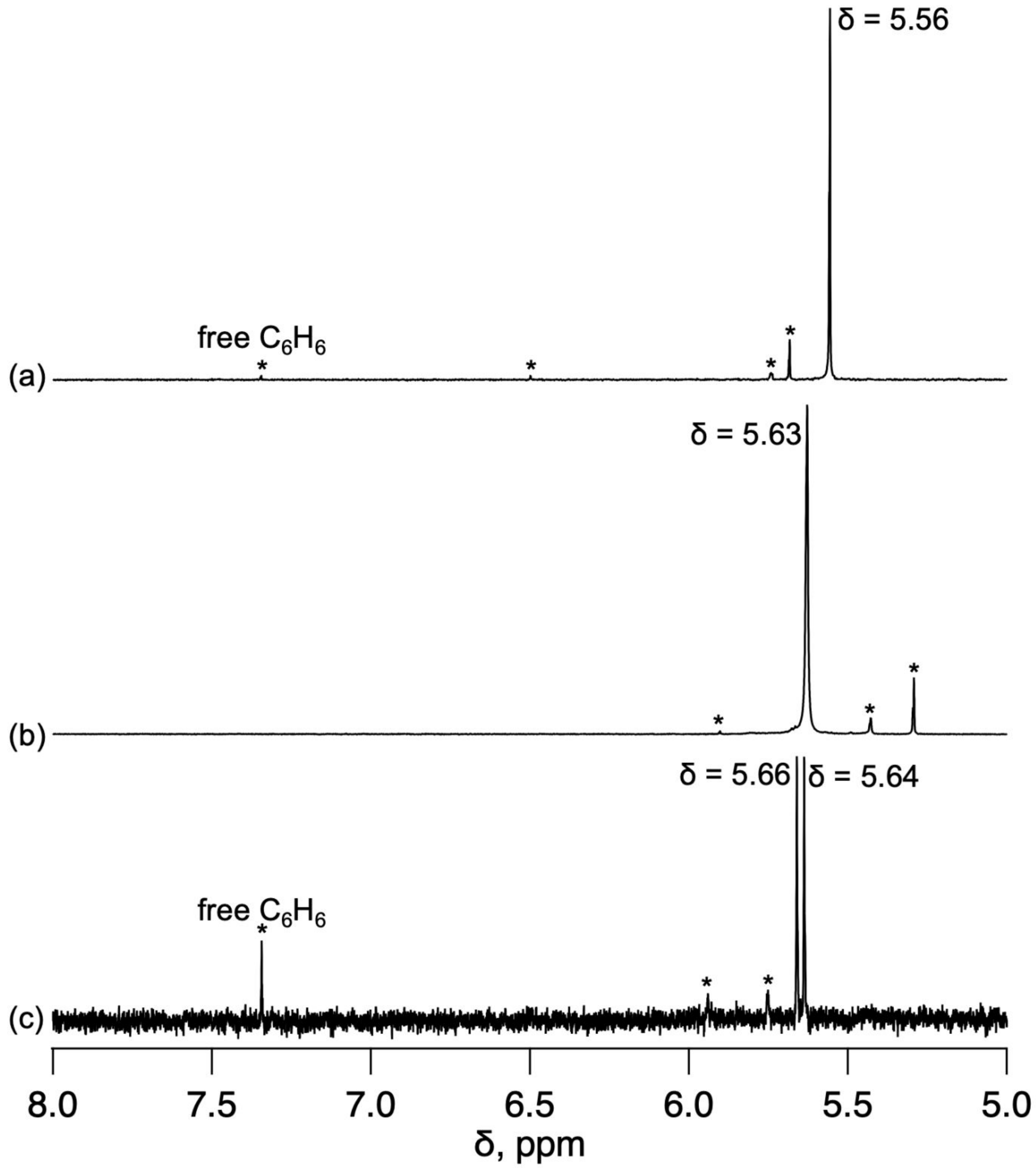

Figure S6. ^{1}H NMR spectra of (a) **1** in $(CD_3)_2SO$, (b) **2** in D_2O, and (c) **3** in $(CD_3)_2SO$. The high noise level in the spectrum of **3** is due to its limited solubility. The residual solvent peak of dimethyl sulfoxide appeared as a multiplet centered at 2.48 ppm. Peaks marked with asterisk sign are due to impurities.

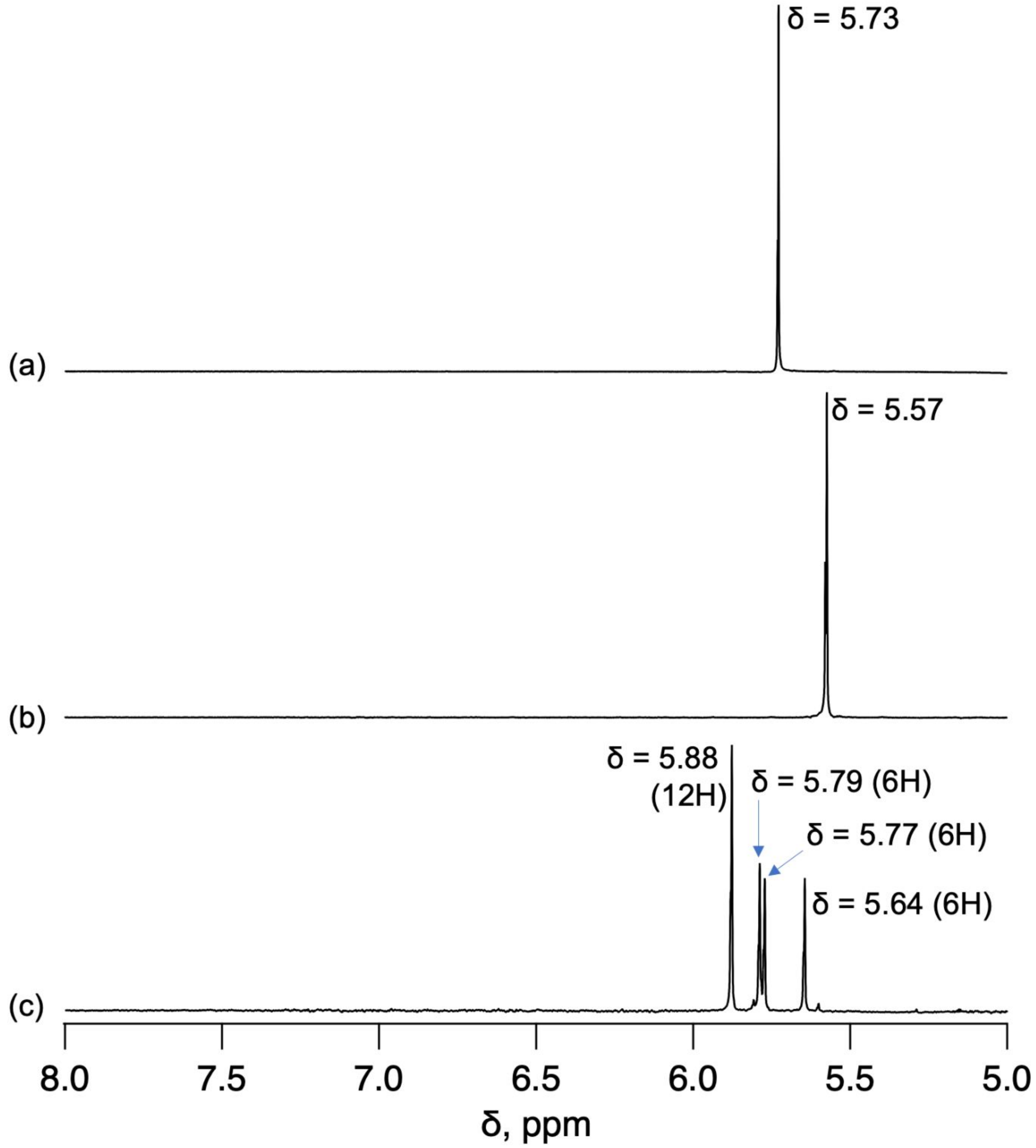

Figure S7. ^1H NMR spectra of (a) **4** in CD_3OD, (b) **5** in D_2O, and (c) **6** in D_2O. In the ^1H NMR spectrum of **6**, the peak at 5.88 ppm corresponds to the benzene ligands attached on Ru3 and Ru4 (see Figure 4a in the original article). The residual solvent peak of methanol appeared as a multiplet centered at 3.30 ppm.

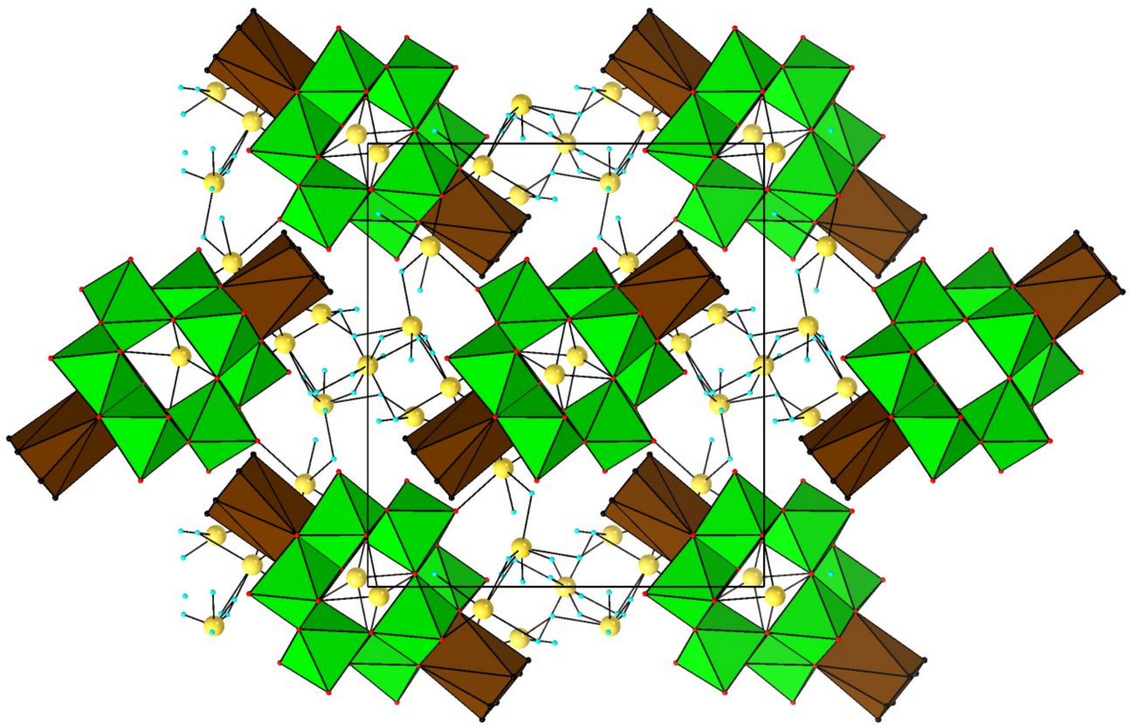

Figure S8. Crystal packing of **5** projected along *a* direction. Tungsten and ruthenium atoms sit inside the green and brown polyhedra, respectively. Yellow spheres are sodium cations. Red and cyan spheres are O^{2-} and the O of water hydrates.

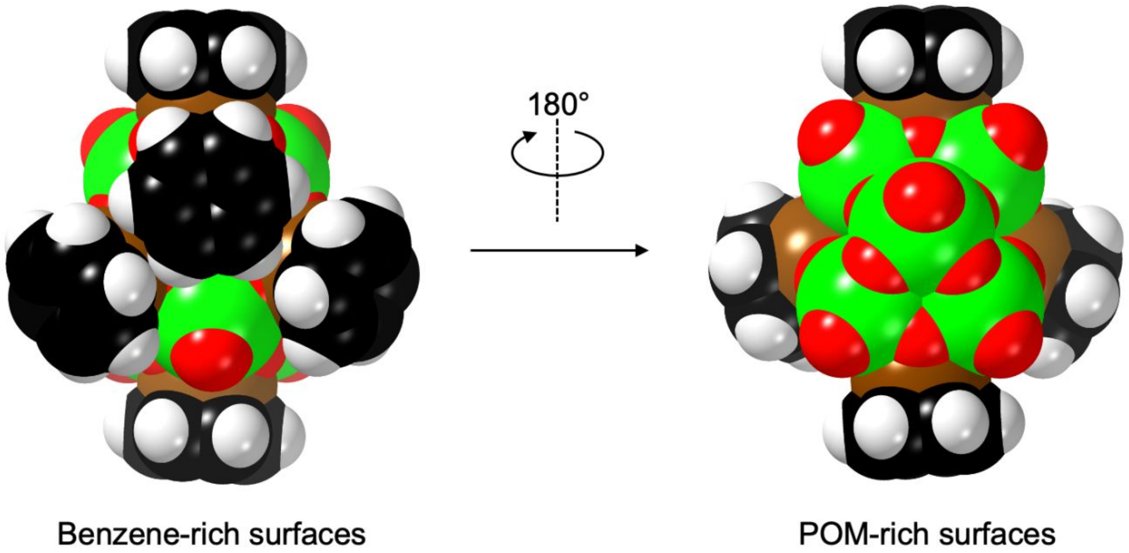

Figure S9. Space filling model of **6** showing the hydrophobic or benzene-rich surfaces and the hydrophilic or POM-rich surfaces. Color scheme: green, W; brown, Ru; red, O; black, C; white, H.

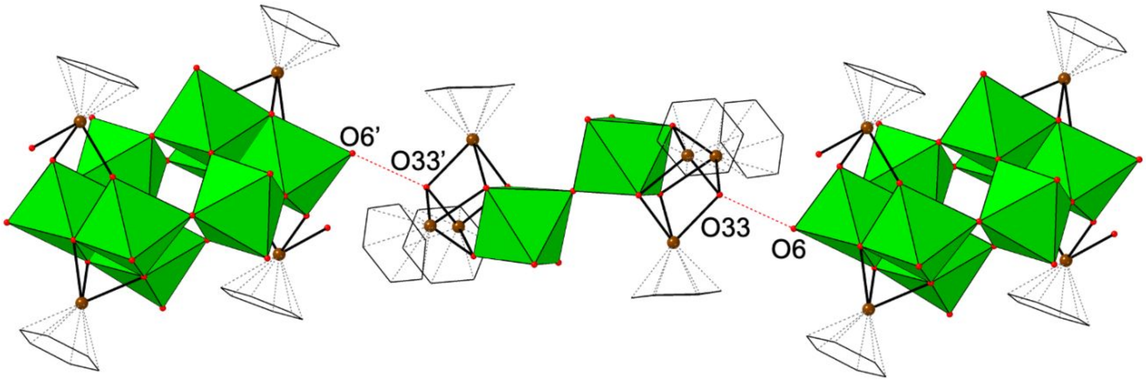

Figure S10. Cation···anion pairs along the [111] direction in **7**. Tungsten atoms sit inside the green octahedra.

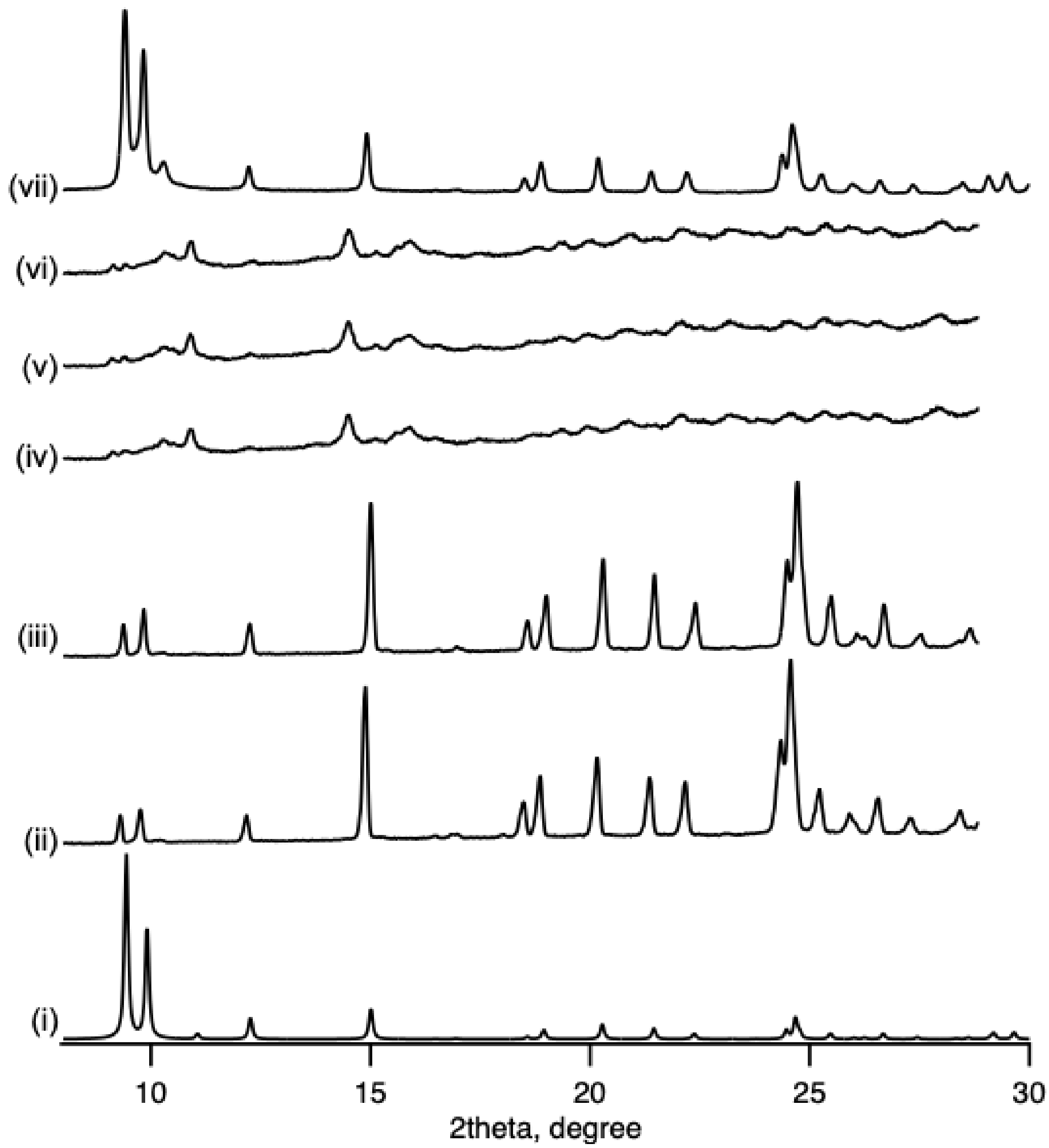

Figure S11. PXRD patterns of (i) **1** simulated using single-crystal data, (ii) **1** measured using a heating device without the flow of nitrogen gas, and (iii)–(vi) **1** measured using a heating device at (trace iii) 30, at (trace iv) 80, at (trace v) 100, and (trace vi) after cooling to 30°C under a flow of nitrogen gas. After the variable-temperature analyses, the sample was exposed to the ambient air for a few minutes, and its PXRD pattern was remeasured (trace vii).

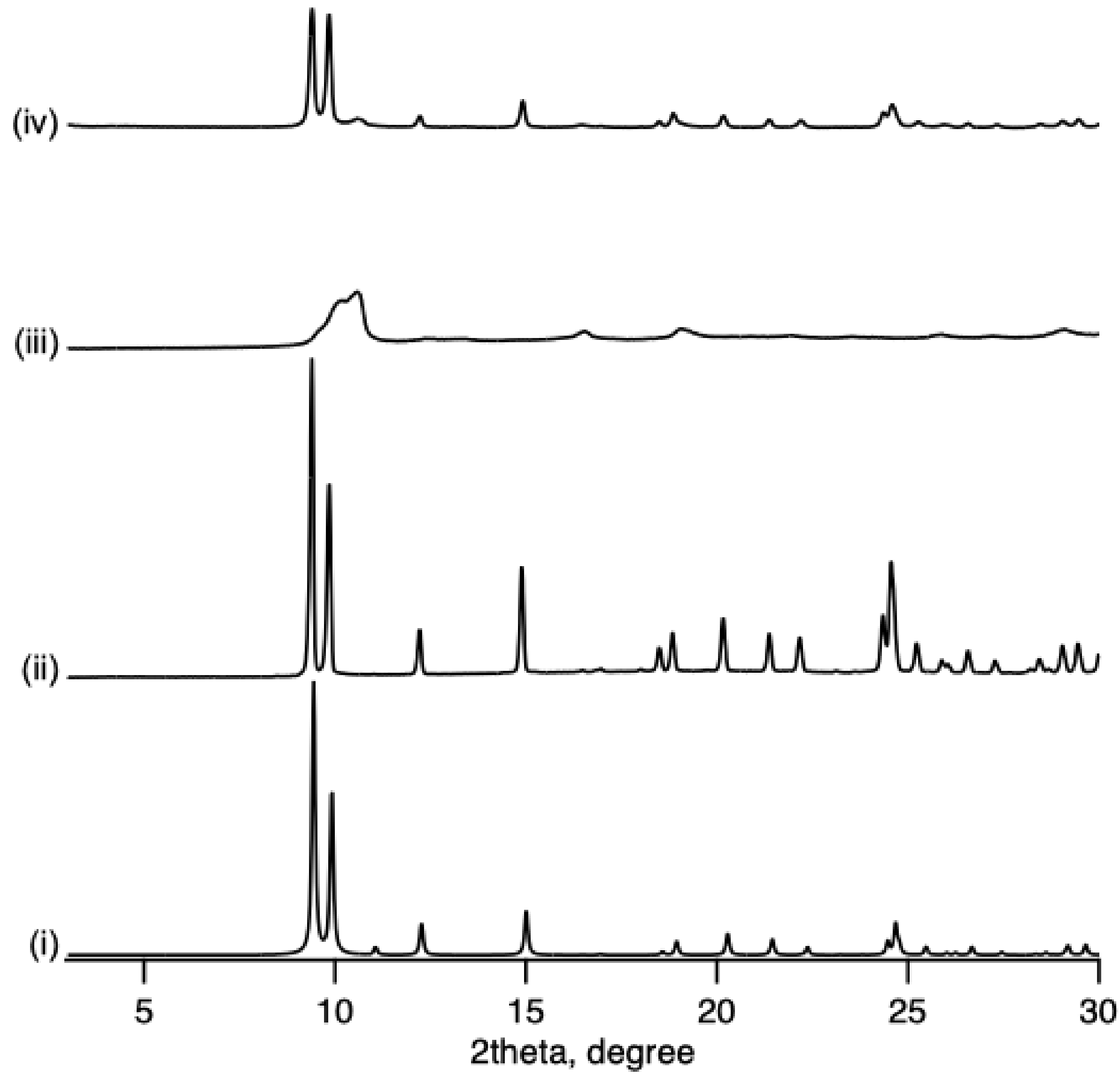

Figure S12. PXRD patterns of (i) **1** simulated using single-crystal data, (ii) **1** in an airtight holder before dehydration, (iii) **1** after dehydration at 100°C under a vacuum condition and sealed in an air-tight holder inside a glovebox. After the measurement, the sample was removed from the cell under the ambient air, and its PXRD pattern was remeasured (trace iv).

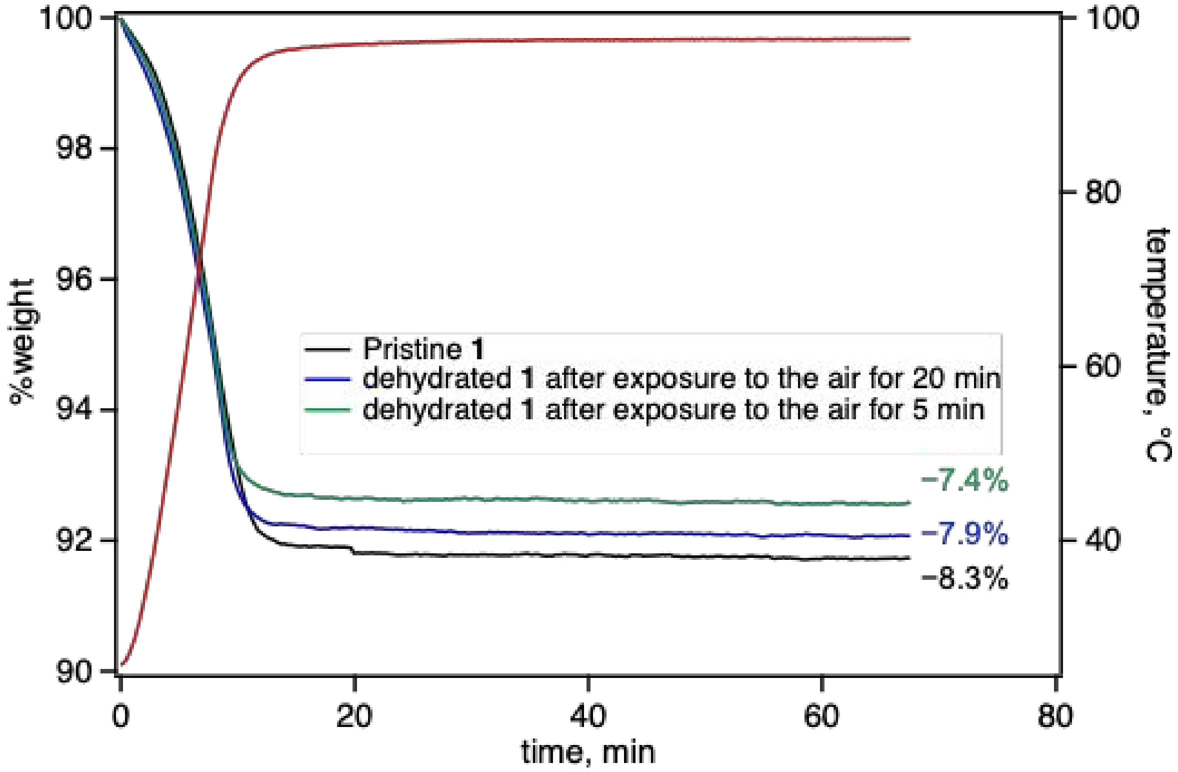

Figure S13. Thermograms showing dehydration and re-hydration of **1** in the air.

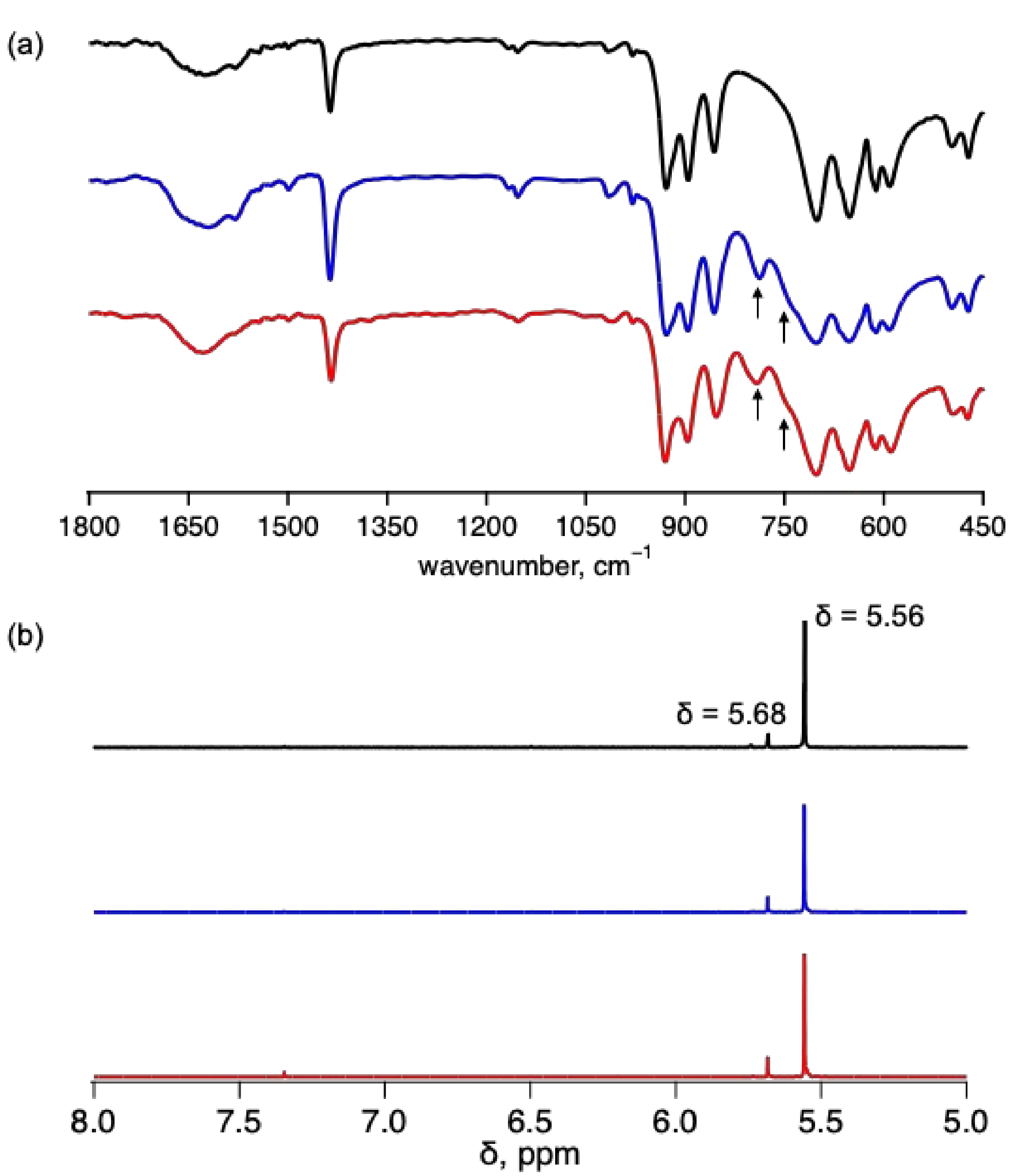

Figure S14. (a) FTIR and (b) ^{1}H NMR spectra of pristine **1** (black traces), **1** after water sorption experiment (blue traces), and **1** after heating in a drying oven at 100°C for 5 d (red traces). Arrows in the FTIR spectra indicate new peaks (789 and 742 cm^{-1}) observed in dehydrated **1**.
